# Rewilding with large herbivores: Positive direct and delayed effects of carrion on plant and arthropod communities

**DOI:** 10.1371/journal.pone.0226946

**Published:** 2020-01-22

**Authors:** Roel van Klink, Jitske van Laar-Wiersma, Oscar Vorst, Christian Smit

**Affiliations:** 1 Groningen Institute for Evolutionary Life Sciences (GELIFES), Conservation Ecology Group, University of Groningen, Groningen, The Netherlands; 2 Independent Researcher, Utrecht, The Netherlands; Universidade de São paulo, BRAZIL

## Abstract

Carrion of large animals is an extremely nutrient rich, ephemeral resource that is essential for many species, but is scarce in the anthropogenic Western-European landscape due to legislative restrictions. Rewilding, a novel conservation strategy that aims at restoring natural processes with minimal human intervention, is increasing in popularity and could lead to increased carrion availability in the landscape. It is therefore important to understand the effects of carrion on biodiversity. We investigated the direct and delayed (five months) effects of red deer (*Cervus elaphus*) carcasses on plants and arthropods in the Oostvaardersplassen, the Netherlands, one of the oldest rewilding sites in Europe. Specifically, we tested whether carrion has a positive direct effect on the abundances and diversity of various arthropod functional groups, as well as a delayed effect on the vegetation and arthropods through the increased nutrient availability. During the active decomposition stage in spring, we, not surprisingly, observed higher abundances of carrion associated species (scavengers and their specialized predators) at the carrion sites than at control sites without carrion, but no higher abundances of predators or detritivores. In late summer, after near-complete decomposition, plant biomass was five times higher, and nutritional plant quality (C:N ratio) was higher at the carrion sites than at the control sites. Arthropod abundance and diversity were also manifold higher, owing to higher numbers of herbivorous and predatory species. Regression analysis showed that abundances of herbivores and detritivores were positively related to plant biomass, and predator abundances were positively related to abundances of herbivores and detritivores, suggesting bottom-up effects propagating through the food chain. Our results show that even in a naturally nutrient-rich ecosystem like the Oostvaardersplassen, carrion can have strong positive effects on local plant biomass and nutritional quality and arthropod abundances, lasting the whole growing season. We found evidence that these effects were first directly caused by the presence of carrion, and later by the enhanced nutrient availability in the soil. This highlights the importance of the indirect pathways by which carrion can structure arthropod communities.

## Introduction

Carrion is a nutrient-rich but ephemeral resource that has become rare in the human dominated landscape of Western Europe. The importance of carrion for obligate scavengers such as vultures or carrion beetles is well recognized, but its importance for the broader fauna remains understudied. Because many vertebrate and invertebrate scavenger species may benefit from carrion directly or indirectly, carrion may significantly contribute to biodiversity [1,2]. The management of many European nature reserves involves grazing by large mammalian herbivores, but since most of these animals are domestic livestock, European legislation requires any dead animals to be removed and destroyed [3,4]. Carcasses of domestic animals are only allowed to remain in the landscape when the aim is to provide food for endangered scavengers such as vultures [5]. The carcasses of culled or hunted wild herbivores are legally allowed to remain *in situ* [4], but such a practice is controversial in many parts of Europe, and has met fierce public opposition [6,7].

With the increasing popularity of rewilding as a nature management strategy, however, the potential carrion pool could increase significantly if carcass disposal regulations can be overcome. Rewilding is a type of conservation management where, usually after reintroduction of some key-stone species (sensu Paine [8]), a non- or minimum intervention policy is practiced in order to maintain self-sustaining, biodiverse ecosystems [9,10]. Higher availability of carrion in nature reserves could be beneficial for carrion associated fauna, as well as for the broader biodiversity, for example due to opportunistic scavenging or increased heterogeneity in nutrient availability to plants. How various trophic groups respond to carrion presence and how long these effects last, however, remains poorly studied.

Carrion enters the ecosystem whenever an animal dies and is not consumed directly by predators. This may occur very frequently (estimates range from 25% to 95% of animal deaths), but varies among ecosystems and species [11,12]. Also kills by predators may become available to scavengers as leftovers, or if the kill is abandoned [11,13,14]. Vertebrate scavengers may detect and utilize such a rich nutrient resource rapidly, especially with warmer weather and when carcasses are large [15]. However, carcasses may also remain undetected by vertebrates [15,16], leading to decomposition being solely performed by invertebrates.

Invertebrates, and particularly insects, on carrion have received considerable attention in the context of forensic entomology (reviewed in [17]) and succession research (reviewed in[18]), but much less in the context of biodiversity research ([1,19], but see[20,21]). Specialized scavenger species, as well as opportunists, which either feed on the carrion itself (e.g. ants), its intestinal content (dung beetles and -flies), or on the scavenging insects (e.g. ants and spiders), colonize fresh carcasses within minutes [22–26]. Opportunistic feeding on carrion and its fluids is not limited to predators and detritivores, but also includes unexpected species such as butterflies, honey bees and true bugs [19,23,24].

During decomposition, a significant amount of the nutrients from a carcass will enter the soil under or near the carcass [27–32], due to leakage of bodily fluids, via excreta of scavengers, and from the intestinal contents of the carcass [33]. After microbial decomposition, these nutrients become available to plants [34]. Plant growth around carrion sites is thus often increased [28,35], and plant nutrient levels are enhanced over ambient conditions [30–32,35,36].

**Fig 1:**
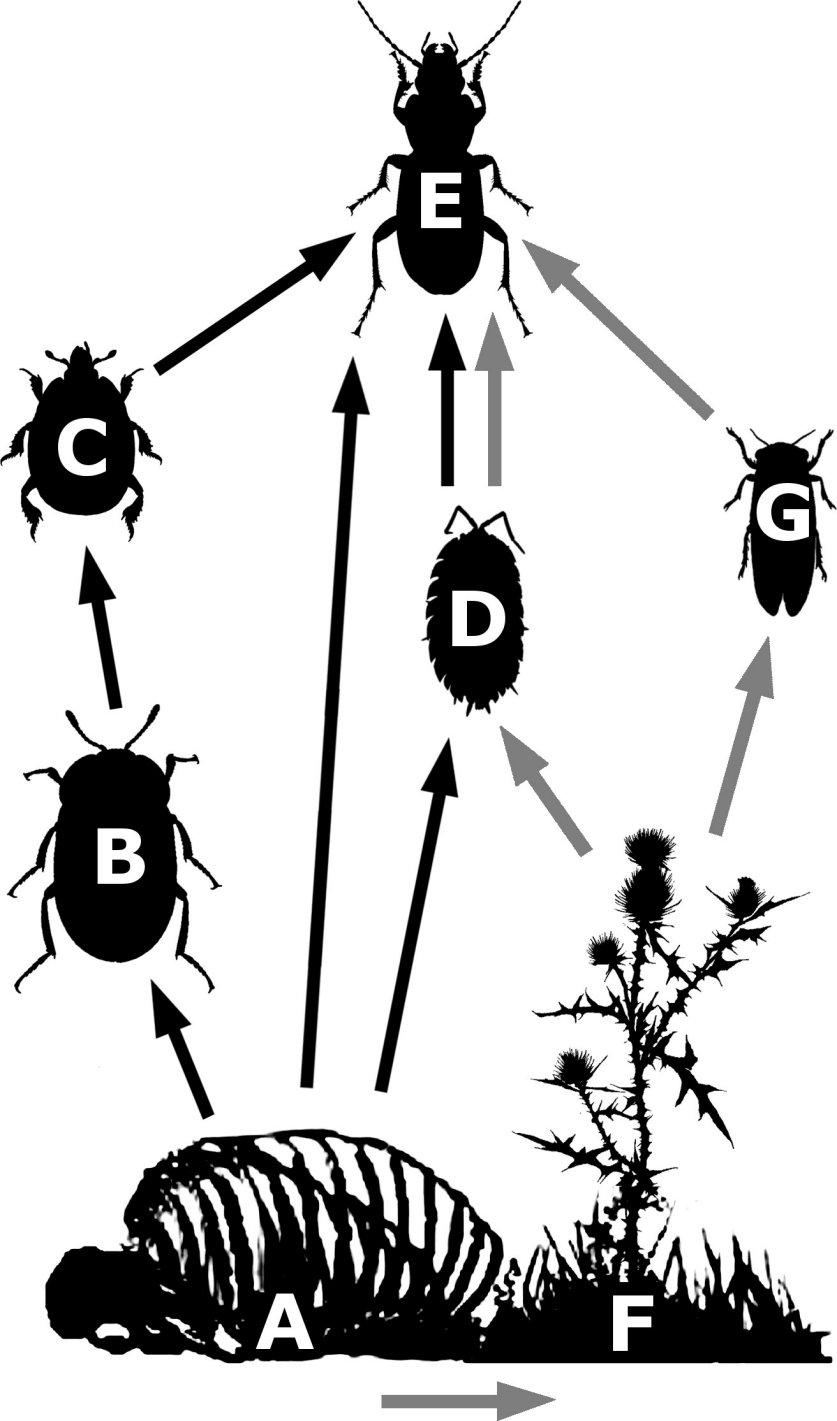
**Hypothesised effects of carrion presence across trophic levels during active decomposition (black arrows) and following complete decomposition (grey arrows).** During decomposition, the carcass (A) will provide resources for obligate scavengers (B) and their specialized predators and parasites (C), as well as facultative scavengers (D and E). Predatory arthropods (E) can also feed on the obligate and facultative scavengers. Following decomposition, plants (F) can use resources leaked into the soil, leading to enhanced populations of herbivorous insects (G). Detritivores (D) can benefit from decaying plant matter and microbial biomass in the soil. Predator (E) abundances can be expected to benefit from these enhanced abundances of herbivores and detritivores.

The resource pulse provided by carrion can thus be expected to propagate across trophic levels, but the pathways may change over the lifetime of a carcass. In this study, we hypothesised that first, invertebrate abundance and species richness will be enhanced through the occurrence of specialized scavengers and their predators (Fig 1 black arrows), as well as opportunistic species. Later, the nutrients from the carcass that entered the soil can result in enhanced plant growth and nutritional value, which can increase the abundance and diversity of herbivorous and detritivorous invertebrates. This should, in turn, enhance abundances of invertebrate predators (Fig 1 grey arrows).

We tested these predictions using red deer (*Cervus elaphus*) carcasses in one of Europe's oldest rewilding sites, the Oostvaardersplassen, the Netherlands. In this reserve, herds of large herbivores, considered wild by law, (Heck cattle (*Bos primigenius taurus*), Konik horses (*Equus ferus caballus*) and red deer) roamed freely in grasslands on nutrient rich clay soils. The introduced large herbivores were exempt from carrion disposal regulations (see [6] for an historical overview of the carrion policy in the Oostvaardersplassen). Due to the absence of top-down control, there tended to be a large die-off of large herbivores at the end of each winter due to food scarcity (up to 30% of the populations), leading to high seasonal carrion availability. Concretely, we expected positive direct effects of carrion on abundances of different functional groups of arthropods in spring, and a delayed effect mediated by vegetation development over the summer.

## Methods

### Study area

Our study was performed in the Oostvaardersplassen (52°26’ N, 5°19’E), a 5600-ha nature reserve situated on reclaimed land, in the province of Flevoland, the Netherlands. The area has a temperate oceanic climate with a mean annual temperature of 9.7°C, and an annual precipitation of on average 833 mm (averages over 1981–2010, data from Royal Netherlands Meteorological Institute www.knmi.nl). The reserve consists of an undrained wetland of 3600 ha (open water and reed beds) and a terrestrial area of 2000 ha. The soil consists of a several meters thick clay layer resting on Pleistocene sands.

Following reclamation in 1969, the area was originally intended for industrial and agricultural use, and partly shortly used for agriculture. It was designated as a nature reserve in the mid 1970’s because of its importance for breeding birds. Three large herbivore species were successively introduced in the area to counteract encroachment by willows (*Salix* spp) and elderberry (*Sambucus nigra*). In 1983, 32 Heck cattle were introduced, followed by 18 Konik horses in 1984, and 52 red deer in 1992–1993. These herds of herbivores grew rapidly and transformed the original shrub cover into a homogeneous grassland in most of the reserve [37–39]. This grassland is best described as a productive, wet grassland on clay soil, dominated by nitrophilous grasses, such as *Lolium perenne* and *Poa trivialis*, and forbs, such as *Sysimbrium officinale*, *Trifolium* sp. and *Carduus crispus*.

Due to the absence of large predators or targeted population control, these populations of large herbivores have increased until 2011, after which a small decrease occurred. At the time of sampling in 2013, there were 155 heads of cattle (0.18 ha^-1^), 800 horses (0.61 ha^-1^) and 1900 red deer (1.8 ha^-1^) in the area, resulting in a total number of 2855 large herbivores (1.4 animals ha^-1^, considering the dry 2000 ha only) [40]. The only management intervention on these populations was an early reactive culling of animals that would not survive the winter, in order to minimize animal suffering [41]. Because the herbivore populations were bottom-up regulated, the highest mortality rates occurred when food was scarcest, in late winter (February–March). The annual mortality rates of the large herbivores reached up to 30% in harsh winters and similar recruitment rates occurred during favourable growing seasons [41]. In the winter preceding sampling, 1728 large herbivores died, of which 1296 red deer [40]. Carcasses of Heck cattle and Koniks were, when accessible, removed by the managers because of public and veterinary concerns, but red-deer carcasses remained in the reserve [6]. Vertebrate scavengers of these carcasses are red fox (*Vulpes vulpes*), raven (*Corvus corax*), carrion crow (*Corvus corone*) and white-tailed eagle (*Hylaeetus albicans*), but because of the high carrion availability, many carcasses remained untouched by vertebrates. The reserve is fenced off from the surrounding agricultural and urban areas, and because the majority of carcasses were left to decompose on site, most nutrients are retained within the reserve boundaries.

### Setup

In late April 2013 five carcasses of adult red deer (healthy live weight >100 kg) were selected in the Oostvaardersplassen. A sample size of five carcasses and five control sites with three pitfall traps each, was chosen because of the expected high number of arthropods to be identified. At this time, carcasses from previous years were only visible as scattered bones in the reserve. The carcasses were at the same stage of decomposition (the advanced decay stage sensu [23]), where most of the flesh, in particular the intestines, had been removed, but carrion associated insects were still present in large numbers (S1 Photo). The carcasses were estimated to be about one month old. Due to the advanced decomposition, the carcasses were left in place. For each carrion site, a matching control site was chosen as the closest site to the carcass where no carrion was present in a 25-m radius, with similar conditions (i.e. presence/absence of dead wood, tree cover, distance from open water, and soil conditions [42]). This was important so that differences in biotic and abiotic conditions would be controlled for. When there was more than one potential control site, the control site was selected at random: first a random direction was determined by twirling a bamboo stick in mid-air and choosing the direction pointed to when it hit the ground. Then, the control site was appointed at the distance of two throws of the stick from the carcass in the direction pointed to. The distance between the carrion and control sites ranged between 37 m and 283 m (mean 100 m). At the start of sampling, there was no visible difference in vegetation structure or composition around the traps, as all vegetation was short-grazed (<5 cm), and annual plants had just started sprouting.

In late August, some five months after death, each site was revisited. At this time, the carcasses had reached the dry stage (sensu [23]), where only skin and bones remain. The two carcasses in the northern part of the reserve could not be found back, so we selected two new sites following the above protocol in the southern part of the reserve. For these newly chosen carcasses, the decomposition stage corresponded with that of the other carcasses (dry stage), although the exact age of the carcasses was unknown. The two new carcasses were in the open with no water or dead wood nearby, and control sites were selected following the method detailed above. At this time, we could detect no difference in plant species richness between the carrion and control sites (GLMM with site and pair as random factors: *P* = 0.61).

### Plant and arthropod sampling and identification

Starting late April, when the carcasses were approximately one month old and the vegetation was short (< 5cm), arthropods were sampled by means of pitfall trapping. We used pitfall traps at this stage of carrion decomposition because we were particularly interested in arthropods migrating to and from the carcasses, as this could be an indication of resource utilisation. At each carcass and control site we placed three pitfall traps (Ø 11 cm, 10 cm deep) to ensure adequate sampling of mobile arthropods. The traps were placed at 1m distance from the carcass or the hypothetical control point (S2 Photo). This distance was chosen to prevent the traps from overflowing with maggots when they migrate from the carcass for pupation. The traps had a coarse mesh cover (3 cm), to prevent capturing small vertebrates, and were covered with rebar-fortified plastic roofs (S2 Photo) for protection against rain and trampling by the large herbivores. The traps contained a 4% formaldehyde solution as preservative to prevent the catch from rotting (which would have attracted more scavengers). The traps were emptied weekly over a six-week period. Because several weekly samples (but not more than 1 week per trap) were lost due to leakage or other causes, we analysed five out of six trapping weeks per trap. From the catch, all ground- and vegetation dwelling arthropods were identified to species level: woodlice (Isopoda), centipedes (Chilopoda), millipedes (Diplopoda), spiders (Araneae), harvestmen (Opiliones), beetles (Coleoptera), true bugs (Heteroptera), plant- and leafhoppers (Auchenorrhyncha), jumping plant lice (Hemiptera: Psylloidea), and earwigs (Dermaptera). Additionally, fly larvae were counted (Diptera), with special attention for blowfly larvae (Calliphoridae). For literature used for identification and nomenclature followed see S1 Table.

Late August, when the carcasses were approximately five months old, each site was revisited to measure the delayed effects on plants and arthropods. Because at this stage we were interested in the local ecosystem impacts of the carcasses, we only sampled the direct vicinity of the carcasses (0–50 cm). Three samples of plants and arthropods were collected at each carcass and control site. This was done by placing a cotton bag with a framed opening of Ø 50cm over the vegetation (to prevent flying insects from escaping) and cutting the vegetation at ground level. Plant litter, detritus and arthropods on the soil surface were then collected with an inverted leaf blower equipped with a fine mesh (< 0,5 mm). At sites with only short vegetation (< 30 cm), suction was applied before cutting of the vegetation to prevent jumping and flying insects from escaping. The same arthropod groups as for the pitfall traps were identified to species level.

### Plant biomass and chemical analysis

In the lab, plant species were separated, dried at 70°C for 24 h and weighed. Four species were sufficiently frequent and provided enough biomass to perform chemical analyses on: *C*. *crispus* (20.8% of biomass), *Plantago major* (4.3%), *Sisymbrium officinale* (29%), *Urtica dioica* (2%) and a mixture of the grass species pooled together (*Lolium perenne*, *Poa annua*, *Poa trivialis* and *Dactylis glomerata*, in total 2.5%). These grass species were pooled for weighing and chemical analysis because they could not all be identified to species level based on the leaves that were damaged by the cutting. *Urtica dioica* was not found at the control sites, therefore five individuals were collected from within 20 m from the control sites where no carcass was present. These individuals were used for the chemical analysis only.

We measured carbon and nitrogen concentrations in the plant tissue of the four most abundant plant species and the pooled grasses. Of all species except *S*. *officinale* we used leaf biomass for chemical analysis, but because *S*. *officinale* yielded insufficient leaf material for chemical analysis, we used the stems of this species. For each carcass or control site, we used one randomly selected sample of each species for measurements. The dried plant matter was ground with a FOSS cyclotec grinder with a 2 mm sieve. We measured the concentrations of elemental C and N according to the DUMAS method, using the Interscience EA 1110 Elemental Analyzer and Eager 200 for Windows. Samples were weighed in tin capsules and brought on a reactor tube with WO_3_ and Cu. After combustion, the N_2_ and CO_2_ were transported through the system, divided by a gaschromatic system and detected with a TCD (thermal conductivity detector). All data collected for this research are available at [43].

### Statistical analysis

#### Classification of arthropod species into functional groups

The identified arthropods were classified into five functional groups: carrion associated fauna, dung associated fauna, predators, herbivores (including pollen feeding beetles and granivores) and non-dung or carrion feeding detritivores (i.e species associated with rotting plant biomass, including fungivores).

Carrion and dung associations were most relevant for beetles, and the species were classified based on the extensive database of beetle catches of the third author. The group of carrion associated species included those known to feed directly on carrion as well as their specialized predators and parasites, and the same approach was taken for dung associations. For both groups, three levels of association were defined: strict association (those species found exclusively in association with carrion or dung), weak association (those species often, but not exclusively found in association with these substrates), and no association. There was a high overlap in the species with weak associations with carrion and dung, and these species may also occur in association with other rotting organic substrates. A summary of functional group attribution at higher taxonomic levels (S1 Text), and the full species list with for each species its carrion or dung association and functional group are provided in the supplementary material (S2 Table).

For all analyses, the group of carrion-associated species consisted of all species with a strict or weak association with carrion. The dung-associated species were those with strict or weak associations with dung, excluding those that are also associated with carrion.

#### Analyses

We tested for differences between the carrion and control sites for arthropod abundances and species richness, and plant biomass and nutrient content using generalized linear mixed models (GLMM). No comparisons were made between the two sampling periods because different sampling methods were used, and because of the replacement of the two lost carcasses. Because the three samples per site and the pairing of carrion and control sites violate the assumption of independent observations, we accounted for this using nested random effects (site nested in pair). We recognize that the p-values resulting from this test should be regarded conservative, therefore we also report the power (1- probability of type ii error) of all tests. We calculated the power using the effect size, standard deviation and sample size (5) of all tests, assuming t-distributions. Arthropod abundance showed overdispersion of the residuals in all cases, therefore we used Penalized Quasi Likelihood (PQL) estimation in Poisson regression [44]. Models for species richness followed a Poisson error structure. We performed a logarithmic (ln) transformation on plant biomass before analysis, to account for heteroscedasticity, and used models assuming a Gaussian distribution of the residuals.

From the measured C and N concentrations in the plant tissue, we calculated C:N ratios and tested for differences between carrion and control sites. To account for heteroscedasticity, the C:N ratios were ln transformed for analysis. Because only one measurement per species per site was taken, only ‘pair’ was included as random factor. To test for differences in C:N ratios across all species, we scaled the C:N ratios of all plant species and ran an LMM of the log-transformed scaled values, with plant species and pair as random factors, to account for non-independence of the measurements. For these tests we again calculated statistical power, using the treatment with the lowest sample size (<5 in case certain plant species were absent at one or more sites).

To test the relations in abundance or biomass between adjacent trophic levels, and whether carrion presence modulated this relationship, we used LMM’s on logarithmically transformed data. For the spring data we regressed predator abundance (excluding carrion associated predators) against the abundance of carrion associated insects (excluding carrion associated predators), and against the abundance of detritivores (excluding carrion and dung associated detritivores). For the summer data, the abundances of herbivores and detritivores (excluding carrion associated detritivores) were regressed against plant biomass, and predator abundance (excluding carrion associated predators) was regressed against the abundances of herbivores and detritivores. In all these models, carrion presence was included as additive fixed effect, but no interactive term was included. These models had the same random structure as above: site nested in pair. All analyses were performed in R 3.4.3 [45].

## Results

In total we collected 18 988 arthropod individuals, which were identified to 366 species. In spring, the most abundant species caught in the pitfall traps were larvae and adults of the carrion beetle *Thanatophilus rugosus* (3702 individuals) and blowfly larvae (4256 individuals)., One of the larvae successfully underwent metamorphosis in captivity, and could be identified to the common with carrion associated species *Cynomya mortuorum*. In summer, most abundant species was the flea beetle *Psylliodes cuprea* (393 individuals), which feeds on Brassicaceae, in this case probably *S*. *officinale*.

### Direct effects

**Fig 2:**
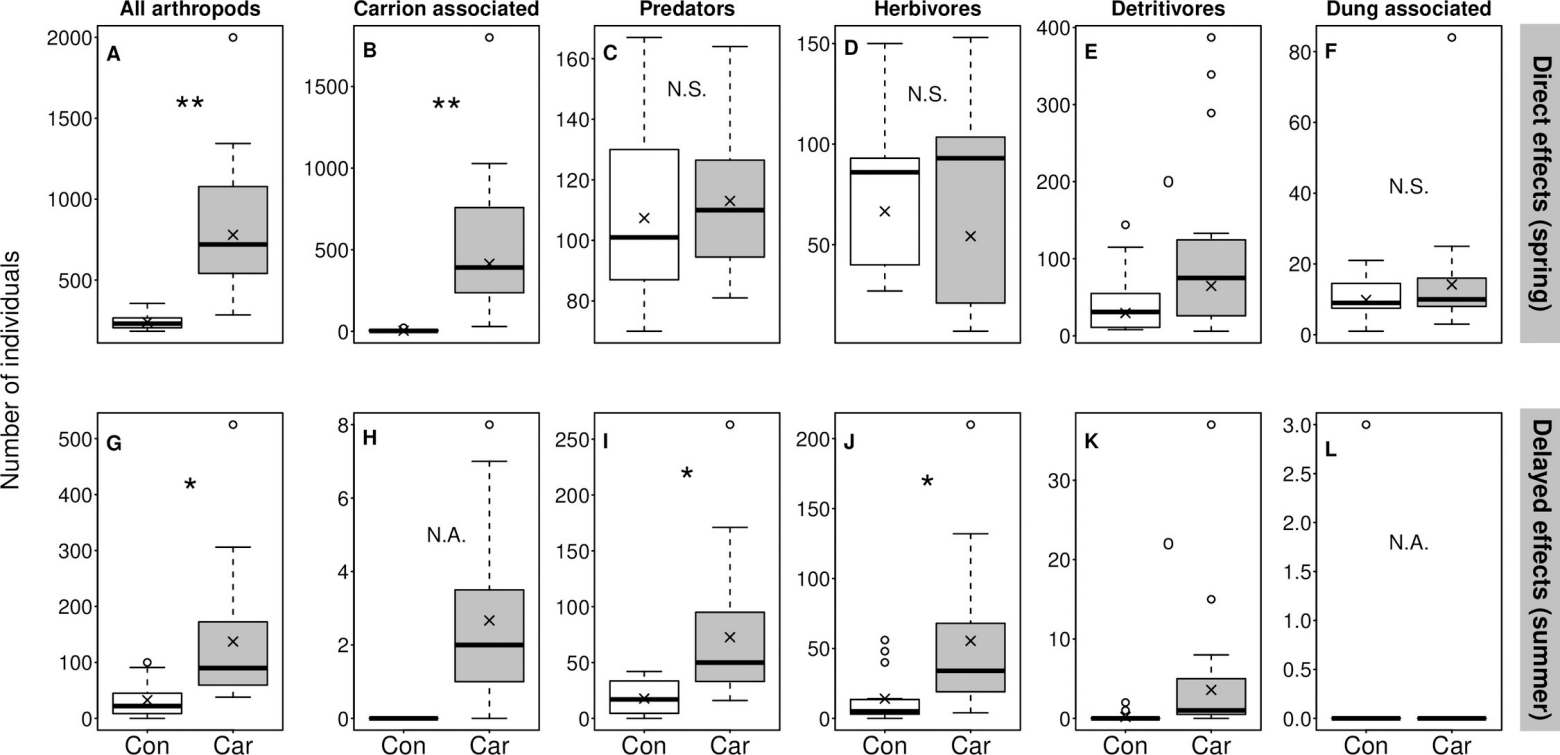
Effect of carrion presence on abundances of arthropods in various trophic groups. Arthropods were sampled in spring, one month after death (top row), and in summer, five months after death (bottom row). Predators, detritivores and dung associated fauna exclude carrion associated species. Crosses represent model estimates, boxes represent the 25% and 75% quartiles from the median (thick line), whiskers represent the maximum and minimum values, excluding outliers, where outliers are defined as points further than 1.5 times the interquartile range. Stars denote significant differences: ** = *P* <0.01; * = *P* < 0.05; ^o^ = P <0.1; N.S. = *P* > 0.1; N.A. = statistical test not possible. ‘Con’ = control sites, ‘Car’ = carrion sites. All test statistics are presented in Table 1.

As expected, during the active decomposition stage, the total number of arthropod individuals was higher (over three times as high) at the carrion sites than at the control sites (Fig 2A; Table 1), but there was no difference in species numbers (z = 0.83; *P* = 0.40). Of the five functional groups, only the carrion associated fauna showed higher abundances at the carrion sites than at the control sites (almost 100 times as many; Fig 2B; t = 6.83, *P* = 0.002), whereas no differences could be detected for any other group (Fig 2C–2F, Table 1). Species richness of the carrion associated fauna at the carrion sites was higher 2.4 times higher (z = 4.70, *P* < 0.001), and 1.4 times higher for the dung-associated fauna (z = 2.08, *P* = 0.038), but did not differ for the other functional groups (all p-values > 0.5; S3 Table).

**Table 1:** Summary statistics of models testing differences in arthropod abundance between carrion and control sites using penalized quasi-likelihood regression. Model convergence failed in cases where in one of the treatments almost only zero counts were present. Significant differences (*P* < 0.05) are denoted bold. Power of the tests is provided to indicate the chance of reporting false negatives.

Panel in Fig 2	Season	Functional group	Estimate	Standard error	t	P-value	Power
A	Spring	All arthropods	1.179	0.196	6.015	**0.004**	0.959
B	Spring	Carrion-associated species	4.548	0.666	6.829	**0.002**	0.987
C	Spring	Predators	0.051	0.136	0.378	0.725	0.056
D	Spring	Herbivores	-0.205	0.204	-1.005	0.372	0.096
E	Spring	Detritivores	0.784	0.365	2.148	0.098	0.268
F	Spring	Dung-associated species	0.368	0.264	1.396	0.235	0.141
G	Summer	All arthropods	1.437	0.382	3.761	**0.020**	0.646
H	Summer	Carrion-associated species	model convergence failed because of excessive zeros				
I	Summer	Predators	1.410	0.327	4.308	**0.013**	0.761
J	Summer	Herbivores	1.369	0.452	3.026	**0.039**	0.469
K	Summer	Detritivores	2.935	1.341	2.188	0.094	0.276
L	Summer	Dung-associated species	model convergence failed because of excessive zeros				

**Fig 3:**
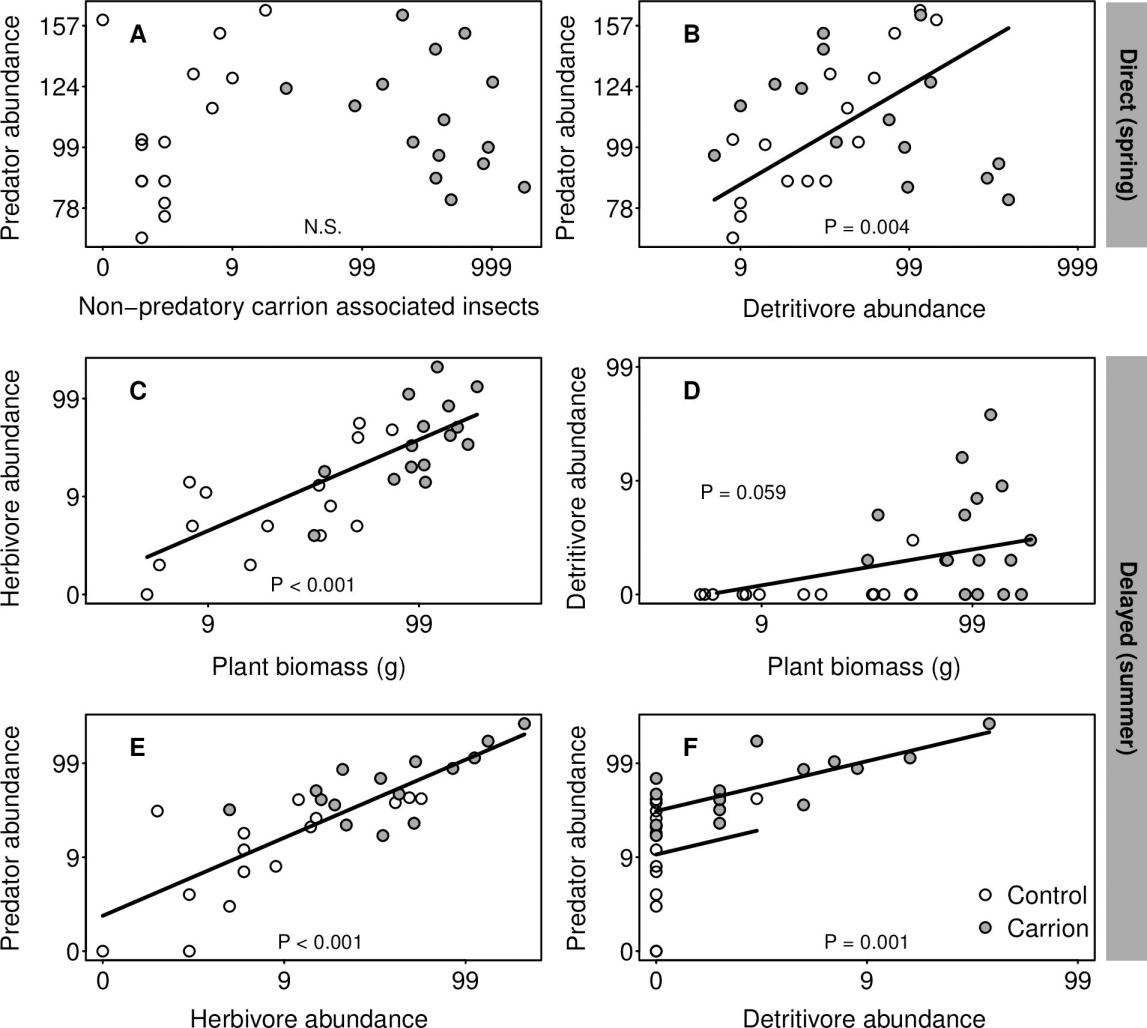
Observed relations between abundances and/or biomass of neighbouring trophic levels. The top row shows the relations in spring, the middle and bottom row show the relations in summer. Note the logarithmic scales of both axes. The relations were fitted using GLMM.

We found no relation between the abundance of carrion associated arthropods (excluding predatory and parasitic species) and non-carrion associated predators (t = 0.38, *P* = 0.71; Fig 3A), but a positive relation between abundances of detritivores and predators (t = 3.21, *P* = 0.004; Fig 3B), while this relation was not affected by carrion presence (*P* = 0.71).

### Delayed effects (summer)

#### Plants

**Fig 4:**
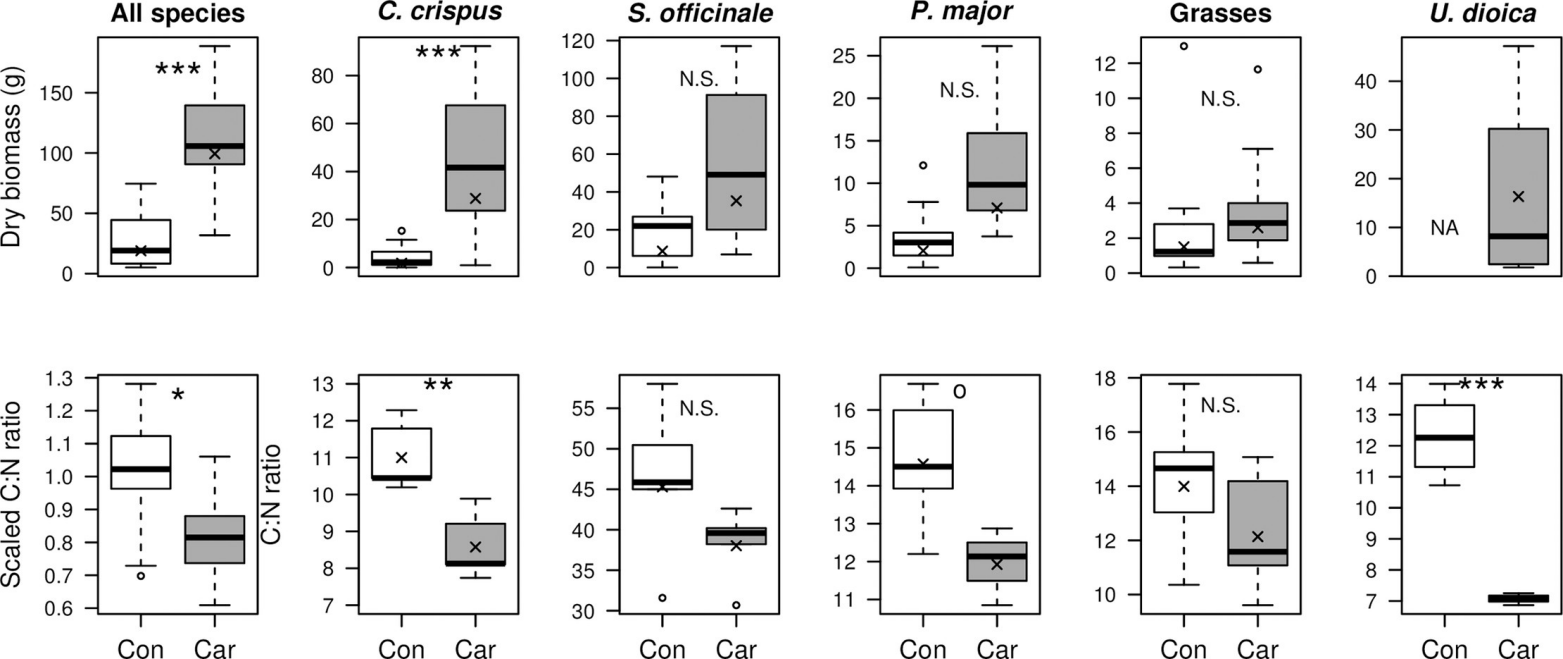
**Vegetation biomass (top row) and nutritional quality (bottom row) responses to carrion presence after five months of decomposition (dry stage).** Nutritional quality was calculated as C:N ratio of leaf or stem material. Crosses represent arithmetic means. Note that because of low power (low sample size) of some tests, large effect sizes do not always provide p-values <0.05. The test statistics and power of all tests are provided in Table 2. Stars denote significant differences: ^o^ = *P* < 0.1; * = *P* < 0.05; ** = *P* <0.01; *** = *P* < 0.001; N.S. = *P* > 0.1. Plant species: *Carduus crispus*, *Sisymbrium officinale*, *Plantago major*, *and Urtica dioica*. ‘Con’ = control sites, ‘Car’ = carrion sites.

In late summer, when the carcasses were almost completely decomposed, substantial differences in vegetation were visible between the carrion and control sites. All carcasses were overgrown with the biannual thistle *Carduus crispus* (S3 Photo). The total plant biomass at the carrion sites was on average five-fold higher than at the control sites (Fig 4; Table 2). This was largely driven by the biomass of *C*. *crispus*, which was significantly higher at carrion sites than control sites (Fig 4, Table 2). For *Sisymbrium officinale*, *Plantago major* and the pooled grasses there was a clear trend towards higher biomass at the carrion sites (i.e. large effect size). However, these statistical tests had low power (Table 2) due to low replication and a relatively large variance, hence, the p-values often exceeded 0.05, despite the clear directional trend (Fig 4 top row).

**Table 2:** Summary statistics of models testing differences in plant biomass and C:N ratio between carrion and control sites. We tested for differences using generalized linear mixed models, which provide conservative p-values. Power of the tests is provided to indicate the chance of reporting false negatives. Significant differences (*P* < 0.05) are denoted bold. Plant species: *Carduus crispus*, *Sisymbrium officinale*, *Plantago major*, *and Urtica dioica*.

	Plant species	Random effects	Estimate	Standard error	t	P-value	Power
Biomass	All species	pair/site	16.573	0.254	6.52	**0.003**	0.98
	*C*. *crispus*	pair/site	27.814	0.551	5.053	**<0.001**	0.88
	*S*. *officinale*	pair/site	1.398	0.791	1.768	0.150	0.2
	*P*. *major*	pair/site	12.362	0.484	2.554	0.207	0.13
	Grasses	pair/site	0.546	0.347	1.573	0.201	0.16
	*U*. *doica*	NA					
C/N ratio	All species	species + pair	-0.247	0.047	-5.251	**<0.001**	0.9
	*C*. *crispus*	pair	-0.249	0.059	-4.205	**0.003**	0.74
	*S*. *officinale*	pair	-0.175	0.115	-1.515	0.168	0.15
	*P*. *major*	pair	-0.201	0.082	-2.446	0.050	0.26
	Grasses	pair	-0.142	0.122	-1.164	0.278	0.11
	*U*. *doica*	pair	-0.55	0.067	-8.2	**<0.001**	0.98

The nutritional quality of the plants was higher at the carcasses than at the control sites, as indicated by lower C:N ratios (Fig 4, Table 2). Again, due to low power, the p-values of some of the species were >0.05, despite relatively large effect sizes (Fig 4 bottom row, Table 2). These tests had invariably low power (Table 2), and hence a high chance of showing false negatives.

#### Arthropods

At the carrion sites, the arthropod abundance was on average 4.2 times higher (Fig 2G, Table 1), and species richness was 2.6 times higher than at the control sites (z = 4.56, *P* < 0.001) in summer. We found fewer carrion-associated arthropod species in summer, despite sampling closer to the carcasses, with only 40 beetle individuals, belonging to nine species, all restricted to the carrion sites (Fig 2H). Because of the high proportion of zero counts at the control sites, no statistical test could be done. The abundance of predators was 4.1 times higher, and the abundance of herbivores was 3.9 times higher at the carrion sites than at control sites (Fig 2I and 2J, Table 1. The abundance of detritivores did not differ significantly between the carrion and control sites (Fig 2K, Table 1). The dung feeding fauna were only represented by three individuals at one of the control sites (Fig 2L). The species richness of predators was 2.7 times higher (z = 7.80, *P* <0.001), herbivore richness was two times higher (z = 2.73 *P* = 0.006), and detritivore richness was five times higher (z = 2.42, *P* = 0.02) at the carrion sites compared to controls (S3 Table).

There was a strong positive relation between plant biomass and the abundance of herbivores (t = 6.41, *P* < 0.001, Fig 3C), and a marginally significant relation with the abundance of detritivores (t = 1.98, *P* = 0.059, Fig 3D), but the presence of carrion did not change these relationships (*P* > 0.05). Predator abundance was positively related to herbivore abundance (t = 7.49, *P* < 0.001, Fig 3E). We found a higher intercept at the carrion sites for the relation between detritivores and predators (carrion: t = 2.63, *P* = 0.044; detritivore abundance: t = 2.40, *P* = 0.030; Fig 3F), which indicates higher abundance of predators at the carrion sites, possibly because of the higher abundance or herbivores (see Fig 2J).

## Discussion

We found clear positive effects of carrion on arthropod abundances and plant biomass. Most striking was the strong positive response of various arthropod groups, visible until at least five months after the death of the large herbivore. The responses differed between the early and late stages of decomposition, from positive direct effects on the carrion associated species, to plant mediated effects in herbivorous and carnivorous species after decomposition was complete. The positive relations within the food web suggest that nutrient-flows from the carcass changed over the decomposition process: from a direct pathway to scavengers, to an indirect pathway through the soil microbes, and plants. By enhancing plant growth near carcasses, carrion may contribute to biotic heterogeneity, which is especially important in a homogeneous, productive former agricultural area such as the Oostvaardersplassen.

Most of the effect sizes were large enough to show strong effects despite the relatively low sample size. For the plant biomass and C:N ratios of some plant species, however, we had low statistical power because of the low sample sizes, which increased the chances of reporting false negatives. A larger sample size would have probably led to p-values < 0.05 also for these species, and can be recommended for future studies. The substitution of two of the carcasses is unlikely to have affected our results, since we did not make a direct comparison between the two sampling periods at each site, but only compared the carrion and control sites at each time.

### Direct effects

The positive and direct effect of carrion on the abundance of arthropod scavengers and their specialised predators and parasites is unsurprising [20,21,46], but we did not detect a direct positive effect of carrion presence on predatory or detritivorous arthropods. We could also not detect a positive relation between the abundances of carrion associated insects and (not carrion-associated) predators, and the weak positive relation between detritivore and predator abundances was independent of carrion presence. This suggests that opportunistic scavenging and predation on carrion associated insects are not important in our system, possibly due to the natural high productivity of the soil, or we failed to detect this for other reasons. In recent work at Yellowstone National Park, only two out of 13 not carrion-associated beetle families (Carabidae and Curculionidae) were found to have higher abundances at carrion than at control sites [21]. Although opportunistic scavenging has often been observed [19,23–26], it thus remains unclear how important this behaviour is for shaping arthropod communities.

How much carrion can directly contribute to biodiversity will depend on the season of death. Mammal and bird diversity at carrion has been reported to be highest in winter [15], whereas, at least in Europe, insect diversity on carrion is highest in spring, [25,47,48]. Decay rates, however, are highest during summer, when temperatures peak [24,25,48]. More generally, it can be expected that in any given ecosystem the availability of carrion is highest after periods of harsh environmental conditions. However, where large predators are present, carrion availability may be more evenly spread throughout the year, which can be more beneficial to scavenger communities.

Scavenger biodiversity can also depend on carcass size. Medium to large carcasses can support more species of both invertebrate [49] and vertebrate scavengers [15,50], but are often monopolized by large vertebrate scavengers [50–52]. Smaller to medium sized carcasses can be beneficial to a broader range of scavenging species, particularly when their availability is unpredictable in time and space [51,52]. Smaller carcasses may even remain undetected by vertebrates [15,16], leaving decomposition completely to invertebrates. Small carcasses, however, decompose quickly, often within a matter of days [23,25,29,53]. The decomposition of larger carcasses may take longer [54,55], and therefore provide a more stable resource to scavenging invertebrates. This may also benefit their predators, such as passerine birds [56]. Thus, the presence of medium to large carcasses in the landscape, such as the red deer carcasses studied here, can contribute positively to the biodiversity of both vertebrates and invertebrates, although the presence of large scavengers may have negative effects on local bird and mammal populations due to enhanced predation [56–58].

### Indirect effects

We found strong positive delayed effects of carrion on the biomass and nutritional contents of plants, and on the abundance of arthropods across trophic levels, in line with our predictions. Such an increase in plant biomass around carrion is likely to be a common phenomenon [32,36,46], but it is rarely measured (but see [28,35]). We recorded these effects some five months after death, but they are likely to last much longer. In other systems, plant biomass was shown to remain elevated up to one year [35], and soil and foliar nutrients up to five [30,31] or even over 10 years after death [36]. It has been suggested that this effect may be (partially) driven by reduced herbivory around carcasses [35,36], but is in our case more likely to be caused by the enhanced nutrient availability provided by the carcass. In fact, grazing by large herbivores at carrion sites may, increase (possibly after a period of avoidance), rather than decrease, due to the availability of nutrient rich forage, despite posing a health risk to the herbivores [35]. In our study area, camera trapping showed that especially Konik horses grazed close to decomposing deer (P. Jansen personal communication), probably driven by food scarcity, which may last until spring. That the enhanced plant growth was driven by nutrient availability is also supported by our finding that the spiny thistle *Carduus crispus* showed an almost tenfold higher biomass at the carrion sites. Because *C*. *crispus* is a biennial species, the overwintering rosette must have been present before the deer died, and was ready to make optimal use of the nutrients from the carcass. This contrasts with the control sites, where the *C*. *crispus* plants remained small.

It stands to reason that carcass size will also strongly determine the delayed responses of vegetation and arthropods, where medium to large carcasses will have much stronger effects than small carcasses, but to our knowledge this has not been tested. Such relations could provide a fruitful avenue for future exploration, and could enhance our understanding of the patch-dynamics of carrion in wild and re-wilded ecosystems significantly.

### Conclusions

Rewilding as a management strategy has gained momentum in nature conservation and academic discourse, but at the moment both the number of rewilding sites and the number of opinion papers on the topic outnumber the publications with actual data [9]. Carrion is a natural part of ecosystems, but legislative restrictions and cultural norms limit the presence of carrion of large mammals in many European nature reserves [6]. In the Oostvaardersplassen, the introduced Heck-cattle and Koniks were exempt from carrion disposal regulations by an exception in legislation, making them legally wild animals [7]. However, even though in the Oostvaardersplassen it is technically legal to leave carcasses of Heck cattle and Koniks to decompose *in situ*, these are usually removed to favour public opinion. During a cold spell in the winter of 2018, public outrage about the starvation of the large herbivores, and in particular the horses in the Oostvaardersplassen reached high levels in the media. It was decided that the herds of large herbivores will be regulated at some 1100 animals, of which 490 red deer[59]. De facto, this meant that the unique bottom-up regulated herbivore population dynamics in this oldest rewilding site will be abandoned. What this means for the carrion availability in the area remains unclear.

Our results show that if carrion regulations can be overcome, even in a naturally productive ecosystem such as the Oostvaardersplassen, large carcasses can significantly increase local invertebrate abundances. The pathways by which carrion affected arthropod communities shifted during the decomposition process, from direct effects on carrion-associated species, to plant and nutrient mediated effects on herbivorous and predatory arthropods. This highlights the importance of indirect pathways by which carrion can structure arthropod communities.

## Supporting information

S1 Photo Red deer carcass undergoing decomposition, ca. 1.5 months after death. (PDF)

S2 Photo Red deer carcass with three pitfall traps, ca. 1.5 months after death. (PDF)

S3 Photo Carrion site some five months after death. (PDF)

S1 Text Summary of functional group attribution. (PDF)

S1 Table Literature used for identification and nomenclature followed for all identified taxa. (PDF)

S2 Table All species found and their functional classification. (PDF)

S3 Table Summary statistic of models testing differences in species numbers between carrion and control sites. (PDF)
