# Supplementary material for: Rewilding with large herbivores: Positive direct and delayed effects of carrion on plant and arthropod communities
Source: PLoS One. 2020 Jan 22;15(1):e0226946. doi: 10.1371/journal.pone.0226946 (PMC6975527; doi:10.1371/journal.pone.0226946)
Supplement: S1 Photo — (PDF) [file pone.0226946.s001.pdf]

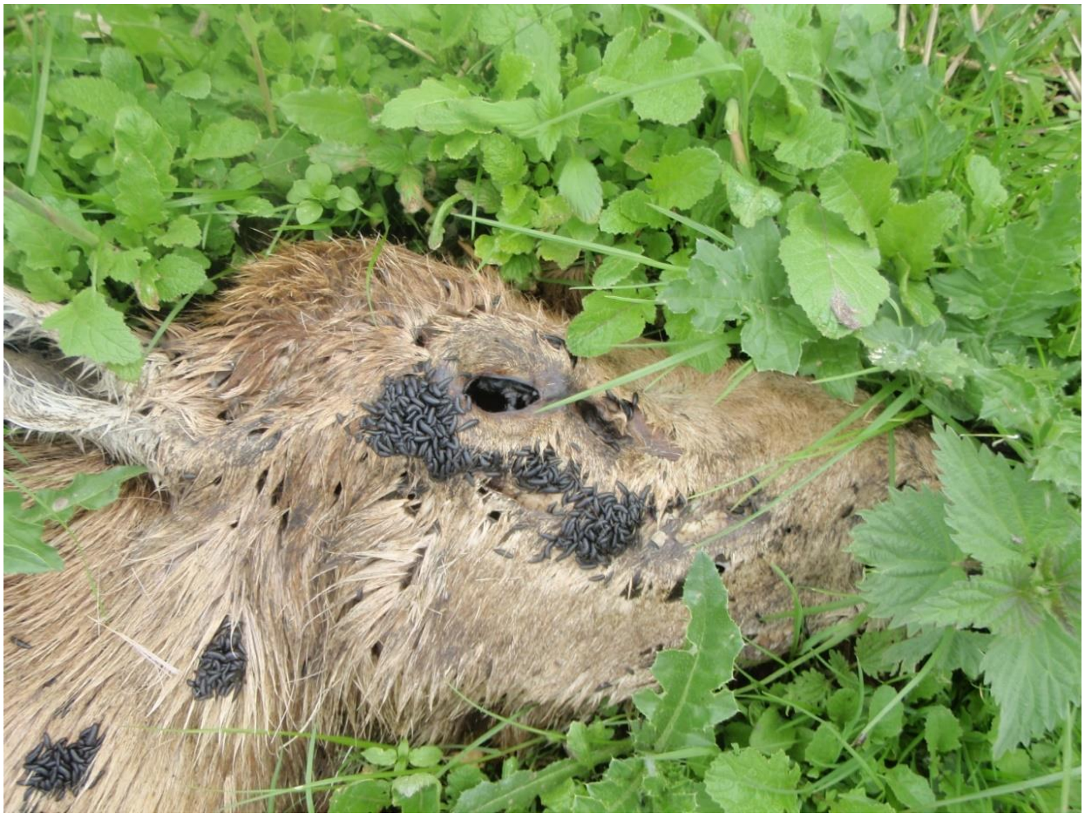

S1 Photo. Red deer carcass undergoing decomposition, ca. 1.5 months after death. The black larvae belong mostly to *Thanatophilus rugosus* (Coleoptera: Silphidae).
