# Supplementary material for: Rewilding with large herbivores: Positive direct and delayed effects of carrion on plant and arthropod communities
Source: PLoS One. 2020 Jan 22;15(1):e0226946. doi: 10.1371/journal.pone.0226946 (PMC6975527; doi:10.1371/journal.pone.0226946)
Supplement: S2 Photo — (PDF) [file pone.0226946.s002.pdf]

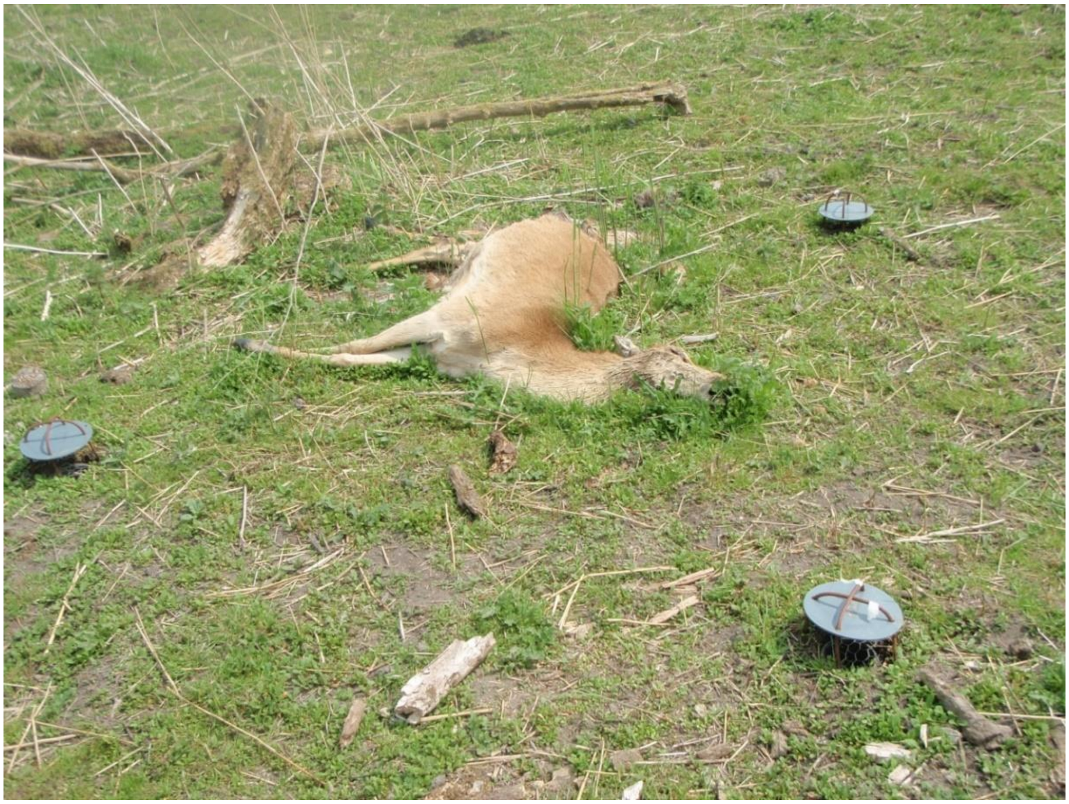

**S2 Photo.** Red deer carcass with three pitfall traps. The traps were covered by rebar fortified roofs, two weeks after placement, May 8th. Same individual as Fig. S1. Photo
