# Supplementary material for: Rewilding with large herbivores: Positive direct and delayed effects of carrion on plant and arthropod communities
Source: PLoS One. 2020 Jan 22;15(1):e0226946. doi: 10.1371/journal.pone.0226946 (PMC6975527; doi:10.1371/journal.pone.0226946)
Supplement: S3 Photo — (PDF) [file pone.0226946.s003.pdf]

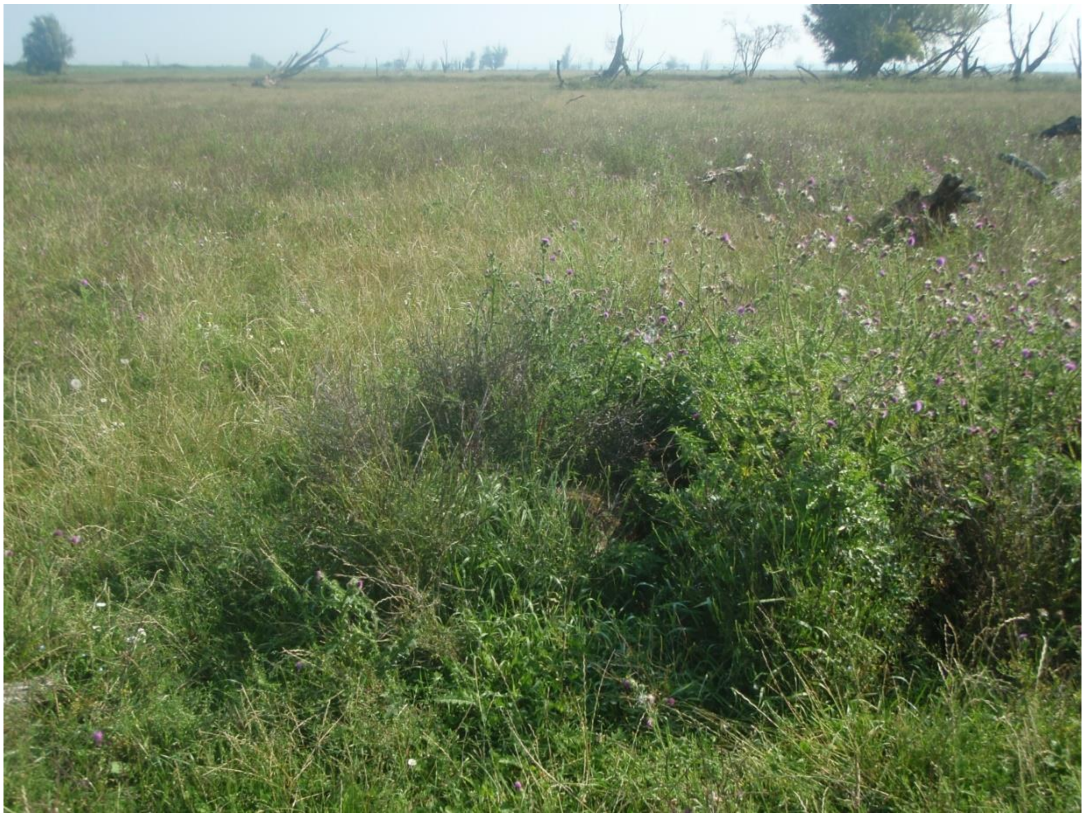

S3 Photo. Carrion site some five months after death. The carcass has reached the dry stage and is surrounded by vigorous plant growth, dominated by the thistle *Carduus crispus*. Photo credit MJJ Schrama
