# Supplementary material for: Rewilding with large herbivores: Positive direct and delayed effects of carrion on plant and arthropod communities
Source: PLoS One. 2020 Jan 22;15(1):e0226946. doi: 10.1371/journal.pone.0226946 (PMC6975527; doi:10.1371/journal.pone.0226946)
Supplement: S1 Text — (PDF) [file pone.0226946.s004.pdf]

S1 Text. Summary of functional group attribution

Carrion associated fauna:

Strict association: Diptera larvae (maggots): Calliphoridae and Sarcophagidae

Coleoptra: Silphidae, Trogidae, Cleridae, Nitidulidae: *Omosita colon*, Histeridae: *Margarinotus brunneus*, Staphylinidae: *Bisnius cephalotes, Creophilus maxilosus, Philontus politus, P. succicola*.

Weak association: Coleoptera Histeridae: *Margarinotus carbonarius*, *M. neglectus*, Staphylinidae: *Aleochara bipustulata, A. lanuginosa, Anotylus sculpturatus, Anotylus tetracarinatus, Atheta atramentaria, Atheta triangulum, Bisnius fimetarius, B. sordidus*, *Gyrophypnus fracticornis, Omalium rivulare, Oxytelus laqueatus, Philontus intermedius, P. splendens, P. varians, Platystethus arenarius, Rugilus rufipes, Tachinus laticollis.*

Dung associated fauna:

Strict association: Coleoptera Scarabaeidae: *Aphodius* sp. and *Onthophagus* sp., Hydrophilidae: *Cercyon* sp., *Cryptopleurum minutum, Sphaeridium* sp., Staphylinidae: *Oxytelus laqueatus, Tachinus laticollis, T. rufipes.*

Weak association: Coleoptera Histeridae: *Margarinotus carbonarius*, *Margarinotus neglectus,* Ptiliidae: *Ptenidium nitidum,* Scarabaeidae*: Oxyomus sylvestris,* Staphylinidae*: Acrotona aterrima , Aleochara bipustulata , A. lanuginosa, Anotylus sculpturatus, A. tetracarinatus, Atheta longicornis, A. triangulum*, *Bisnius fimetarius*, *Omalium rivulare, Oxypoda acuminata, Philonthus intermedius, P. laminatus, P. splendens, P. varians.*

Predators:

Araneae and Opiliones, Chilopoda, Staphylinidae not associated with carrion or dung, some Carabidae, Cantharidae, Coccinellidae, Histeridae: *Kissister minimus*, Heteroptera: Anthocoridae, Nabidae, Saldidae, Miridae: *Chalamidatus*

Herbivores:
Auchenorrhyncha, Psylloidea, Coleoptera: Brentidae, Chrysomelidae, Elateridae, Curculionidae, Kateretidae, Scarptiidae, Nitidulidae (excl *Omosita*), Phalacridae, Pyrochroidae, Heteroptera: Lygaeidae, Miridae, excl *Chlamidatus saltitans*, Tingidae, larvae of Diptera Tephritidae and Lepidoptera living in the flower heads of *Carduus crispus*.

Detritivores (all species depending on dead organic matter, other than carrion and dung, incl. species feeding on fungi):
Isopoda, Diplopoda, Dermaptera, Coleoptera: Clambidae, Corylophidae, Cryptophagidae, Hydrophylidae, Hydraenidae, Latridiidae, Leiodidae, Monotomidae, Ptiliidae, Scirtidae.
