# Supplementary material for: Rewilding with large herbivores: Positive direct and delayed effects of carrion on plant and arthropod communities
Source: PLoS One. 2020 Jan 22;15(1):e0226946. doi: 10.1371/journal.pone.0226946 (PMC6975527; doi:10.1371/journal.pone.0226946)
Supplement: S1 Table — (PDF) [file pone.0226946.s005.pdf]

S1 Table. Literature used for identification and nomenclature followed for all identified taxa.

Taxon	Identification	Nomenclature
Isopoda	Berg & Wijnhoven (1997)	Berg & Wijnhoven (1997)
Chilopoda	Berg & Evenhuis (2001)	Berg & Evenhuis (2001)
Diplopoda	Hauser & Voigtländer (2009)	Hauser & Voigtländer (2009)
Araneae	Roberts (1987)	World Spider Catalog (2018)
Opiliones	Wijnhoven (2009)	Wijnhoven (2009)
Dermaptera		
Heteroptera	Stichel (1955 - 1962)	Aukema & Rieger (1995-2006)
Auchenorrhyncha	Biedermann & Niedringhaus (2003)	Biedermann & Niedringhaus (2003)
Sternorrhyncha: Psyllidae	Ossiannilsson (1992)	Psyl'list (Ouvrard, 2017)
Coleoptera: Carabidae	Muilwijk (2015)(preprint)	Vorst ed. (2010)
Other Coleoptera	Freude et al. (1964-2012)	Vorst ed. (2010)

Aukema, B., Rieger, C., 1995. Catalogue of the Heteroptera of the Palearctic region 1-6. Nederlandse Entomologische Vereniging, Amsterdam.

Berg, M.P., Evenhuis, C., 2001. Determinatietabel voor de Nederlandse duizendpoten (Myriapoda: Chilopoda). Ned. Faun. Meded. 15, 41–78.

Berg, M.P., Wijnhoven, H., 1997. Landpissebedden - Een tabel voor de landpissebedden (Crustacea; Oniscidae) van Nederland en België. KNNV Uitgeverij, Utrecht.

Biederman, R., Niedringhaus, R., 2003. Die Zikaden Deutschlands. W.A.B.V. Fründ, Scheeßel.

Freude, H., Harde, K.W., Lohse, G.A., Klausnitzer, B. (eds), n.d. Die Käfer Mitteleuropas bd 1-15. Goecke & Everts, Krefeld.

Hauser, H., Voigtländer, K., 2009. Doppelfüßer (Diplopoda) Ostdeutschlands. Deutscher Jugendbund für Naturbeobachtung, Göttingen.

Muilwijk, J., Felix, R., Dekoninck, W., Bleich, O., 2015. De loopkevers van Nederland en België (Carabidae). Ned. Faun. Meded. Suppl. 9, 215 pp.

Ossiannilsson, F., 1992. The Psylloidea (Homoptera) of Fennoscandia and Denmark. Brill, Leiden, New York, Köln.

Ouvrard, D., 2017. Psyl'list - The World Psylloidea Database. [WWW Document]. doi:10.5519/0029634

Roberts, M.J., 1987. The Spiders of Great Britain and Ireland. Harley books, Colchester.

Stichel, W., 1955 - 1962. Illustrierte Bestimmungstabellen der Wanzen. II. Europa. (Hemiptera - Heteroptera Europae). Vols 1 - 4. Stichel, Berlin-Hermsdorf.

Vorst, O., 2010. Catalogus van de Nederlandse kevers (Coleoptera). Monografiën van de Nederlandse Entomologische Vereniging 11. Nederlandse Entomologische Vereniging, Amsterdam.

Wijnhoven, H., 2009. De Nederlandse Hooiwagens (Opiliones). Entomologische Tabellen 3. Ned. Faun. Meded. Suppl. 3, 118 pp.

World Spider Catalog, 2018. World Spider Catalog Version 19.0. Natural History Museum Bern [WWW Document]. URL http://www.wsc.nmbe.ch/ (accessed February 4 18).
