# Supplementary material for: Rewilding with large herbivores: Positive direct and delayed effects of carrion on plant and arthropod communities
Source: PLoS One. 2020 Jan 22;15(1):e0226946. doi: 10.1371/journal.pone.0226946 (PMC6975527; doi:10.1371/journal.pone.0226946)
Supplement: S2 Table — (PDF) [file pone.0226946.s006.pdf]

S2 Table. All species found and their functional classification. Trophic classes: detr: detritivorous, herb = herbivorous, pred = predatory, ? = unknown. Trophic class within carrion and dung associations: carrion = feeding on the carrion, carrionpara = parasitic on carrion associated insects, larv_pred = larvae predatory on dung or carrion, pred = predatory on dung or carrion associated insects, dung = feeding on dung, fungi = fungivore on dung or carrion, detr = weak carrion association, general detritivore.

Class / order / suborder	Family	Species	Spring	Summer	Carrion assoc.	Dung assoc.	Trophic class	Trophic class for dung/ carrion association
Crustacea								
Isopoda	Philosciidae	*Philoscia muscorum* (Scopoli, 1763)	24	0	none	none	detr	
Isopoda	Porcellionidae	*Porcellio scaber* Latreille, 1804	61	0	none	none	detr	
Isopoda	Trachelipodidae	*Trachelipus rathkii* (Brandt, 1833)	4	0	none	none	detr	
Isopoda	Trichoniscidae	*Trichoniscus pusillus* Brandt, 1833	84	0	none	none	detr	
Myriapoda								
Chilopoda	Lithobiidae	*Lithobius forficatus* (Linnaeus, 1758)	41	2	none	none	pred	
Diplopoda	Craspedosomatidae	*Craspedosoma rawlinsii* Leach, 1815	43	1	none	none	detr	
Diplopoda	Julidae	*Brachyiulus pusillus* (Leach, 1815)	1639	0	none	none	detr	
Diplopoda	Julidae	*Cylindroiulus britannicus* (Verhoeff, 1891)	12	0	none	none	detr	
Diplopoda	Julidae	*Julus scandinavicus* Latzel, 1884	42	0	none	none	detr	
Diplopoda	Polydesmidae	*Brachydesmus superus* (Latzel, 1884)	44	0	none	none	detr	
Diplopoda	Polydesmidae	*Polydesmus denticulatus* C.L.Koch, 1847	10	0	none	none	detr	
Diplopoda	Polydesmidae	*Polydesmus inconstans* Latzel, 1884	17	0	none	none	detr	
Diplopoda	Polydesmidae	Female and juveline *Polydesmus*	48	0	none	none	detr	
Arachnida								
Araneae	Araneidae	*Larinioides cornutus* (Clerck, 1757)	0	10	none	none	pred	
Araneae	Clubionidae	*Clubiona phragmitis* C. L. Koch, 1843	3	3	none	none	pred	
Araneae	Clubionidae	*Clubiona* sp.	2	17	none	none	pred	
Araneae	Corinnidae	*Phrurolithus festivus* (C. L. Koch, 1835)	1	0	none	none	pred	
Araneae	Gnaphosidae	*Micaria pulicaria* (Sundevall, 1831)	3	0	none	none	pred	
Araneae	Linyphiidae	*Bathyphantes approximatus* (O. P.-Cambridge, 1871)	1	0	none	none	pred	
Araneae	Linyphiidae	*Bathyphantes gracilis* (Blackwall, 1841)	122	14	none	none	pred	
Araneae	Linyphiidae	*Centromerita bicolor* (Blackwall, 1833)	5	1	none	none	pred	
Araneae	Linyphiidae	*Collinsia inerrans* (O. P.-Cambridge, 1885)	11	7	none	none	pred	
Araneae	Linyphiidae	*Dicymbium nigrum* (Blackwall, 1834)	14	0	none	none	pred	

Araneae	Linyphiidae	*Diplocephalus cristatus* (Blackwall, 1833)	1	0	none	none	pred
Araneae	Linyphiidae	*Diplocephalus latifrons* (O. P-Cambridge, 1863)	50	1	none	none	pred
Araneae	Linyphiidae	*Diplostyla concolor* (Wider, 1834)	63	2	none	none	pred
Araneae	Linyphiidae	*Dismodicus bifrons* (Blackwall, 1841)	5	0	none	none	pred
Araneae	Linyphiidae	*Erigone atra* Blackwall, 1833	55	16	none	none	pred
Araneae	Linyphiidae	*Erigone dentipalpis* (Wider, 1834)	26	4	none	none	pred
Araneae	Linyphiidae	*Agyneta rurestris* (C. L. Koch, 1836)	7	2	none	none	pred
Araneae	Linyphiidae	*Mermessus trilobatus* (Emerton, 1882)	3	0	none	none	pred
Araneae	Linyphiidae	*Neriene montana* (Clerck, 1757)	5	0	none	none	pred
Araneae	Linyphiidae	*Oedothorax agrestis* (Blackwall, 1853)	1	0	none	none	pred
Araneae	Linyphiidae	*Oedothorax apicatus* (Blackwall, 1850)	1	3	none	none	pred
Araneae	Linyphiidae	*Oedothorax fuscus* (Blackwall, 1834)	31	2	none	none	pred
Araneae	Linyphiidae	*Oedothorax gibbosus* (Blackwall, 1841)	1	0	none	none	pred
Araneae	Linyphiidae	*Oedothorax retusus* (Westring, 1851)	2	0	none	none	pred
Araneae	Linyphiidae	*Porrhomma microphthalmum* (O. P.-Cambridge, 1871)	5	0	none	none	pred
Araneae	Linyphiidae	*Porrhomma oblitum* (O. P.-Cambridge, 1871)	6	0	none	none	pred
Araneae	Linyphiidae	*Tenuiphantes tenuis* (Blackwall, 1852)	34	19	none	none	pred
Araneae	Linyphiidae	*Tiso vagans* (Blackwall, 1834)	6	1	none	none	pred
Araneae	Linyphiidae	*Troxochrus scabriculus* (Westring, 1851)	51	33	none	none	pred
Araneae	Linyphiidae	*Walckenaeria nudipalpis* (Westring, 1851)	2	0	none	none	pred
Araneae	Linyphiidae	Juvenile Linyphiidae	6	529	none	none	pred
Araneae	Lycosidae	*Alopecosa pulverulenta* (Clerck, 1757)	1	0	none	none	pred
Araneae	Lycosidae	*Pardosa agrestis* (Westring, 1861)	1	0	none	none	pred
Araneae	Lycosidae	*Pardosa amentata* (Clerck, 1757)	409	0	none	none	pred
Araneae	Lycosidae	*Pardosa palustris* (Linnaeus, 1758)	2	0	none	none	pred
Araneae	Lycosidae	*Pardosa prativaga* (L. Koch, 1870)	27	0	none	none	pred
Araneae	Lycosidae	*Pardosa pullata* (Clerck, 1757)	1	0	none	none	pred
Araneae	Lycosidae	*Piratula hygrophila* (Thorell, 1872)	12	0	none	none	pred
Araneae	Lycosidae	*Tapinocyba insecta* (L. Koch, 1869)	1	0	none	none	pred
Araneae	Lycosidae	*Trochosa ruricola* (De Geer, 1778)	7	0	none	none	pred
Araneae	Lycosidae	Juvenile Lycosidae	9	0	none	none	pred
Araneae	Mimetidae	*Ero cambridgei* Kulczyński, 1911	2	0	none	none	pred
Araneae	Tetragnathidae	*Pachygnatha clercki* Sundevall, 1823	85	8	none	none	pred
Araneae	Tetragnathidae	*Pachygnatha degeeri* Sundevall, 1830	53	10	none	none	pred
Araneae	Tetragnathidae	*Tetragnatha extensa* (Linnaeus, 1758)	0	3	none	none	pred
Araneae	Therididae	*Enoplognatha ovata* (Clerck, 1757)	1	0	none	none	pred
Araneae	Therididae	*Robertus arundineti* (O. P.-Cambridge, 1871)	1	0	none	none	pred
Araneae	Therididae	*Robertus lividus* (Blackwall, 1836)	6	0	none	none	pred
Araneae	Thomisidae	*Ozyptila praticola* (C. L. Koch, 1837)	2	1	none	none	pred
Araneae	Thomisidae	*Xysticus lanio* C. L. Koch, 1835	0	1	none	none	pred
Araneae	Thomisidae	*Xysticus ulmi* (Hahn, 1831)	3	0	none	none	pred

Araneae	Thomisidae	Juvenile *Xysticus*	0	2	none	none	pred
Opiliones	Phalangiidae	*Mitopus morio* (Fabricius, 1799)	2	1	none	none	pred
Opiliones	Phalangiidae	*Oligolophus tridens* (C.L. Koch, 1836)	5	0	none	none	pred
Opiliones	Phalangiidae	*Rilaena triangularis* (Herbst, 1799)	31	0	none	none	pred
<u>Insecta</u>							
Dermaptera	Forficulidae	*Forficula auricularia* Linnaeus, 1758	3	12	none	none	detr
Heteroptera	Anthocoridae	*Anthocoris nemorum* (Linnaeus, 1761)	0	73	none	none	pred
Heteroptera	Anthocoridae	*Orius majusculus* (Reuter, 1879)	0	220	none	none	pred
Heteroptera	Anthocoridae	*Orius minutus* (Linnaeus, 1758)	0	8	none	none	pred
Heteroptera	Anthocoridae	*Orius niger* (Ribaut, 1923)	0	28	none	none	pred
Heteroptera	Anthocoridae	*Orius vicinus* (Wolff, 1811)	0	1	none	none	pred
Heteroptera	Lygaeidae	*Lygus rugulipennis* Poppius, 1911	0	7	none	none	herb
Heteroptera	Miridae	*Apolygus spinolae* (Meyer-Dür, 1841)	1	0	none	none	herb
Heteroptera	Miridae	*Chlamydatus saltitans* (Fallén, 1807)	0	10	none	none	pred
Heteroptera	Miridae	*Liocoris tripustulatus* (Fabricius, 1781)	0	1	none	none	herb
Heteroptera	Miridae	*Lygocoris pabulinus* (Linnaeus, 1761)	0	3	none	none	herb
Heteroptera	Miridae	*Notostira elongata* (Geoffroy, 1785)	0	1	none	none	herb
Heteroptera	Miridae	*Scolopostethus affinis*(Schilling, 1829)	2	3	none	none	herb
Heteroptera	Miridae	*Scolopostethus thomsoni* Reuter, 187	1	0	none	none	herb
Heteroptera	Miridae	*Trigonotylus caelestialium* (Kirkaldy, 1902	0	3	none	none	herb
Heteroptera	Nabidae	*Nabis ferus* (Linnaeus, 1758)	0	2	none	none	pred
Heteroptera	Saldidae	*Saldula orthochila* (Fieber, 1859)	1	5	none	none	pred
Heteroptera	Tingidae	*Tingis ampliata* (Herrich-Schaeffer, 1838)	8	0	none	none	herb
Heteroptera	Tingidae	*Tingis cardui* (Linnaeus, 1758)	0	2	none	none	herb
Auchenorrhyncha	Cicadellidae	*Anoscopus flavostriatus* (Donovan, 1799)	0	3	none	none	herb
Auchenorrhyncha	Cicadellidae	*Aphrodes makarovi* Zachvatkin, 1948	0	11	none	none	herb
Auchenorrhyncha	Cicadellidae	*Arthaldeus pascuellus* (Fallen, 1826)	0	3	none	none	herb
Auchenorrhyncha	Cicadellidae	*Deltocephalus pulicaris (Fallen, 1806)*	0	4	none	none	herb
Auchenorrhyncha	Cicadellidae	*Eupteryx aurata* (Linnaeus, 1758)	0	2	none	none	herb
Auchenorrhyncha	Cicadellidae	*Eupteryx urticae* (Fabricius, 1803)	0	1	none	none	herb
Auchenorrhyncha	Cicadellidae	Juvenile Cicadellidae	1	0	none	none	herb
Auchenorrhyncha	Cicadellidae	Juvenile *Macrosteles*	0	1	none	none	herb
Auchenorrhyncha	Cicadellidae	Juvenile *Typhlocybinae*	0	2	none	none	herb
Auchenorrhyncha	Cicadellidae	*Macrosteles cristatus* (Ribaut, 1927)	0	6	none	none	herb
Auchenorrhyncha	Cicadellidae	*Streptanus aemulans* (Kirschbaum, 1868)	0	5	none	none	herb
Auchenorrhyncha	Cicadellidae	*Streptanus sordidus* (Zetterstedt, 1828)	0	65	none	none	herb
Auchenorrhyncha	Cicadellidae	*Zyginidia scutellaris* (Herrich-Schäffer, 1838)	1	0	none	none	herb

Auchenorrhyncha	Delphacidae	*Javesella dubia* (Kirschbaum, 1868)	2	64	none	none	herb
Auchenorrhyncha	Delphacidae	*Javesella pelucida* (Fabricius, 1794)	1	0	none	none	herb
Auchenorrhyncha	Delphacidae	Juvenile *Delphacidae*	0	5	none	none	herb
Sternorrhyncha	Psyllidae	*Cacopsylla ambigua* (Foerster, 1848)	1	0	none	none	herb
Coleoptera	Brentidae	*Ceratapion gibbirostre* (Gyllenhal, 1813)	6	1	none	none	herb
Coleoptera	Brentidae	*Ceratapion onopordi* (Kirby, 1808)	36	0	none	none	herb
Coleoptera	Brentidae	*Ischnopterapion virens* (Herbst, 1797)	28	52	none	none	herb
Coleoptera	Brentidae	*Protapion fulvipes* (Geoffroy, 1785)	8	11	none	none	herb
Coleoptera	Cantharidae	*Cantharis fusca* Linnaeus, 1758	10	0	none	none	pred
Coleoptera	Cantharidae	*Cantharis rufa* Linnaeus, 1758	1	0	none	none	pred
Coleoptera	Cantharidae	*Malthodes europaeus* (Wittmer, 1970)	5	0	none	none	pred
Coleoptera	Cantharidae	*Rhagonycha fulva* (Scopoli, 1763)	1	0	none	none	pred
Coleoptera	Carabidae	*Acupalpus exiguus* Dejean, 1829	3	0	none	none	pred
Coleoptera	Carabidae	*Agonum fuliginosum* (Panzer, 1809)	1	0	none	none	pred
Coleoptera	Carabidae	*Agonum muelleri* (Herbst, 1784)	3	0	none	none	pred
Coleoptera	Carabidae	*Amara aenea* (De Geer, 1774)	28	0	none	none	herb
Coleoptera	Carabidae	*Amara aulica* (Panzer, 1796)	4	0	none	none	herb
Coleoptera	Carabidae	*Amara bifrons* (Gyllenhal, 1810)	0	2	none	none	herb
Coleoptera	Carabidae	*Amara familiaris* (Duftschmidt, 1812)	16	11	none	none	herb
Coleoptera	Carabidae	*Amara ovata* (Fabricius, 1792)	4	0	none	none	herb
Coleoptera	Carabidae	*Amara similata* (Gyllenhal, 1810)	24	0	none	none	herb
Coleoptera	Carabidae	*Anchomenus dorsalis* (Pontoppidan, 1763)	130	2	none	none	pred
Coleoptera	Carabidae	*Badister bullatus* (Schrank, 1798)	2	0	none	none	pred
Coleoptera	Carabidae	*Badister sodalis* (Duftschmidt, 1812)	42	0	none	none	pred
Coleoptera	Carabidae	*Bembidion aeneum* Germar, 1824	5	0	none	none	pred
Coleoptera	Carabidae	*Bembidion biguttatum* (Fabricius, 1779)	42	0	none	none	pred
Coleoptera	Carabidae	*Bembidion guttula* (Fabricius, 1792)	4	0	none	none	pred
Coleoptera	Carabidae	*Bembidion lampros* (Herbst, 1784)	18	1	none	none	pred
Coleoptera	Carabidae	*Bembidion obtusum* Audinet-Serville, 1821	3	0	none	none	pred
Coleoptera	Carabidae	*Bembidion properans* (Stephens, 1828)	25	0	none	none	pred
Coleoptera	Carabidae	*Bembidion quadrimaculatum* (Linnaeus, 1760)	2	0	none	none	pred
Coleoptera	Carabidae	*Bembidion tetracolum* Say, 1823	2	0	none	none	pred
Coleoptera	Carabidae	*Calathus rotundicollis* Dejean, 1828	1	0	none	none	pred
Coleoptera	Carabidae	*Carabus granulatus* (Linnaeus, 1758)	270	0	none	none	pred
Coleoptera	Carabidae	*Chlaenius nigricornis* (Fabricius, 1787)	1	0	none	none	pred
Coleoptera	Carabidae	*Clivina fossor* (Linnaeus, 1758)	16	0	none	none	pred
Coleoptera	Carabidae	*Harpalus affinis* (Schrank, 1781)	50	0	none	none	herb
Coleoptera	Carabidae	*Harpalus rufipes* (De Geer, 1774)	159	2	none	none	herb
Coleoptera	Carabidae	*Loricera pilicornis* (Fabricius, 1775)	4	0	none	none	pred

Coleoptera	Carabidae	*Nebria brevicollis* (Fabricius, 1792)	5	0	none	none	pred	
Coleoptera	Carabidae	*Notiophilus aquaticus* (Linnaeus, 1758)	0	1	none	none	pred	
Coleoptera	Carabidae	*Notiophilus substriatus* Waterhouse, 1833	2	1	none	none	pred	
Coleoptera	Carabidae	*Oxypselaphus obscurus* (Herbst, 1784)	15	0	none	none	pred	
Coleoptera	Carabidae	*Poecilus cupreus* (Linnaeus, 1758)	10	0	none	none	pred	
Coleoptera	Carabidae	*Pterostichus melanarius* (Illiger, 1798)	4	0	none	none	pred	
Coleoptera	Carabidae	*Pterostichus niger* (Schaller, 1783)	12	0	none	none	pred	
Coleoptera	Carabidae	*Pterostichus nigrita* (Paykull, 1790)	2	0	none	none	pred	
Coleoptera	Carabidae	*Pterostichus oblongopunctatus* (Fabricius, 1787)	5	0	none	none	pred	
Coleoptera	Carabidae	*Pterostichus strenuus* (Panzer, 1796)	220	3	none	none	pred	
Coleoptera	Carabidae	*Pterostichus vernalis* (Panzer, 1796)	63	0	none	none	pred	
Coleoptera	Carabidae	*Syntomus truncatellus* (Linnaeus, 1760)	1	0	none	none	pred	
Coleoptera	Carabidae	*Trechus obtusus* Erichson, 1837	3	1	none	none	pred	
Coleoptera	Chrysomelidae	*Aphthona euphorbiae* (Schrank, 1781)	1	0	none	none	herb	
Coleoptera	Chrysomelidae	*Cassida flaveola* Thunberg, 1794	10	2	none	none	herb	
Coleoptera	Chrysomelidae	*Cassida rubiginosa* Müller, 1776	3	3	none	none	herb	
Coleoptera	Chrysomelidae	*Chaetocnema concinna* (Marsham, 1802)	7	67	none	none	herb	
Coleoptera	Chrysomelidae	*Chaetocnema hortensis* (Geoffroy, 1785)	1	1	none	none	herb	
Coleoptera	Chrysomelidae	*Chrysolina fastuosa* (Scopoli, 1763)	34	0	none	none	herb	
Coleoptera	Chrysomelidae	*Chrysolina graminis* (Linnaeus, 1758)	10	0	none	none	herb	
Coleoptera	Chrysomelidae	*Crepidodera plutus* (Latreille, 1804)	3	0	none	none	herb	
Coleoptera	Chrysomelidae	*Gastrophysa polygoni* (Linnaeus, 1758)	0	4	none	none	herb	
Coleoptera	Chrysomelidae	*Longitarsus kutscherae* (Rye, 1872)	0	2	none	none	herb	
Coleoptera	Chrysomelidae	*Longitarsus luridus* (Scopoli, 1763)	3	0	none	none	herb	
Coleoptera	Chrysomelidae	*Longitarsus melanocephalus* (De Geer, 1775)	22	5	none	none	herb	
Coleoptera	Chrysomelidae	*Longitarsus parvulus* (Paykull, 1799)	2	0	none	none	herb	
Coleoptera	Chrysomelidae	*Phyllotreta nemorum* (Linnaeus, 1758)	74	9	none	none	herb	
Coleoptera	Chrysomelidae	*Phyllotreta nigripes* (Fabricius, 1775)	2	0	none	none	herb	
Coleoptera	Chrysomelidae	*Phyllotreta undulata* Kutschera, 1860	14	22	none	none	herb	
Coleoptera	Chrysomelidae	*Psylliodes chalcomera* (Illiger, 1807)	36	3	none	none	herb	
Coleoptera	Chrysomelidae	*Psylliodes cuprea* (Koch, 1803)	408	393	none	none	herb	
Coleoptera	Clambidae	*Clambus armadillo* (De Geer, 1774)	6	0	none	none	detr	
Coleoptera	Cleridae	*Necrobia violacea* (Linnaeus, 1758)	2	6	strict	none		carrion
Coleoptera	Coccinellidae	*Coccidula rufa* (Herbst, 1783)	0	1	none	none	pred	
Coleoptera	Coccinellidae	*Coccinella septempunctata* Linnaeus, 1758	0	3	none	none	pred	
Coleoptera	Coccinellidae	*Harmonia axyridis* (Pallas, 1773)	0	1	none	none	pred	
Coleoptera	Corylophidae	*Corylophus cassidoides* (Marsham, 1802)	4	1	none	none	detr	
Coleoptera	Corylophidae	*Sericoderus lateralis* (Gyllenhal, 1827)	0	1	none	none	detr	
Coleoptera	Cryptophagidae	*Atomaria apicalis* Erichson, 1846	0	1	none	none	detr	
Coleoptera	Cryptophagidae	*Atomaria basalis* Erichson, 1846	26	0	none	none	detr	
Coleoptera	Cryptophagidae	*Atomaria fuscata* (Schönherr, 1808)	5	1	none	none	detr	

Coleoptera	Cryptophagidae	*Atomaria rubella* Heer, 1841	38	48	none	none	detr	
Coleoptera	Cryptophagidae	*Atomaria scutellaris* Motschulsky, 1849	6	3	none	none	detr	
Coleoptera	Cryptophagidae	*Atomaria testacea* Stephens, 1830	2	4	none	none	detr	
Coleoptera	Cryptophagidae	*Ephistemus globulus* (Paykull, 1898)	0	4	none	none	detr	
Coleoptera	Curculionidae	*Amalus scortillum* (Herbst, 1795)	14	10	none	none	herb	
Coleoptera	Curculionidae	*Ceutorhynchus chalybaeus* Germar, 1824	66	54	none	none	herb	
Coleoptera	Curculionidae	*Ceutorhynchus erysimi* (Fabricius, 1787)	373	31	none	none	herb	
Coleoptera	Curculionidae	*Ceutorhynchus pallidactylus* (Marsham, 1802)	1	0	none	none	herb	
Coleoptera	Curculionidae	*Ceutorrhynchus pyrrhorhynchus* (Marsham, 1802)	0	54	none	none	herb	
Coleoptera	Curculionidae	*Ceutorrhynchus typhae* (Herbst, 1795)	22	36	none	none	herb	
Coleoptera	Curculionidae	*Hadroplontus litura* (Fabricius, 1775)	5	0	none	none	herb	
Coleoptera	Curculionidae	*Hypera nigrirostris* (Fabricius, 1775)	6	6	none	none	herb	
Coleoptera	Curculionidae	*Nedyus quadrimaculatus* (Linnaeus, 1758)	17	0	none	none	herb	
Coleoptera	Curculionidae	*Pelenomus quadrituberculatus* (Fabricius, 1787)	76	65	none	none	herb	
Coleoptera	Curculionidae	*Phyllobius pomaceus* Gyllenhal, 1834	8	0	none	none	herb	
Coleoptera	Curculionidae	*Rhinoncus inconspectus* (Herbst, 1795)	1	0	none	none	herb	
Coleoptera	Curculionidae	*Rhinoncus pericarpius* (Linnaeus, 1758)	3	0	none	none	herb	
Coleoptera	Curculionidae	*Rhinoncus perpendicularis* (Reich 1797)	0	4	none	none	herb	
Coleoptera	Curculionidae	*Sitona hispidulus* (Fabricius, 1777)	59	2	none	none	herb	
Coleoptera	Curculionidae	*Sitona lepidus* Gyllenhal, 1834	104	6	none	none	herb	
Coleoptera	Curculionidae	*Tanymecus palliatus* (Fabricius, 1787)	1	0	none	none	herb	
Coleoptera	Curculionidae	*Tanysphyrus lemnae* (Paykull, 1792)	1	0	none	none	herb	
Coleoptera	Curculionidae	*Trichosirocalus troglodytes* (Fabricius, 1787)	3	0	none	none	herb	
Coleoptera	Elateridae	*Agriotes accuminatus* (Stephens, 1830)	17	0	none	none	herb	
Coleoptera	Elateridae	*Agriotes lineatus* (Linnaeus, 1767)	114	0	none	none	herb	
Coleoptera	Elateridae	*Agriotes obscurus* (Linnaeus, 1758)	252	0	none	none	herb	
Coleoptera	Elateridae	*Agriotes sputator* (Linnaeus, 1758)	34	0	none	none	herb	
Coleoptera	Elateridae	*Ampedus balteatus* (Linnaeus, 1758)	1	0	none	none	?	
Coleoptera	Elateridae	*Athous haemorrhoidalis* (Fabricius, 1801)	1	0	none	none	herb	
Coleoptera	Elateridae	*Dalopius marginatus* (Linnaeus, 1758)	2	0	none	none	herb	
Coleoptera	Elateridae	*Denticollis linearis* (Linnaeus, 1758)	3	0	none	none	?	
Coleoptera	Histeridae	*Hister bisexstriatus* Fabricius, 1801	2	0	none	none	pred	
Coleoptera	Histeridae	*Kissister minimus* (Laporte, 1840)	110	4	none	none	pred	
Coleoptera	Histeridae	*Margarinotus brunneus* (Fabricius, 1775)	91	0	strict	none		carrionpara
Coleoptera	Histeridae	*Margarinotus carbonarius* (Hoffmann, 1803)	2	3	weak	weak		carrionpara
Coleoptera	Histeridae	*Margarinotus neglectus* (Germar, 1813)	1	0	weak	weak		carrionpara
Coleoptera	Histeridae	*Margarinotus purpurascens* (Herbst, 1791)	1	0	none	none	pred	carrionpara
Coleoptera	Hydrophilidae	*Cercyon impressus* (Sturm, 1807)	4	0	none	strict		larv_pred
Coleoptera	Hydrophilidae	*Cercyon lateralis* (Marsham, 1802)	3	0	none	strict		larv_pred
Coleoptera	Hydrophilidae	*Cercyon melanocephalus* (Linnaeus, 1757	18	1	none	strict		larv_pred
Coleoptera	Hydrophilidae	*Cercyon pygmaeus* (Illiger, 1801)	2	0	none	strict		larv_pred

Coleoptera	Hydrophilidae	*Cryptopleurum minutum* (Fabricius, 1775)	3	2	none	strict		larv_pred
Coleoptera	Hydrophilidae	*Helophorus aequalis* Thomson, 1868	1	2	none	none	larv_pred	
Coleoptera	Hydrophilidae	*Helophorus brevipalpis* Bedel, 1881	40	3	none	none	larv_pred	
Coleoptera	Hydrophilidae	*Helophorus grandis* Illiger, 1798	1	0	none	none	larv_pred	
Coleoptera	Hydrophilidae	*Helophorus minutus* (Fabricius, 1775	1	0	none	none	larv_pred	
Coleoptera	Hydrophilidae	*Megasternum concinnum* (Marsham, 1802)	29	18	none	none	larv_pred	
Coleoptera	Hydrophilidae	*Sphaeridium* cf. *scarabaeoides* (Linnaeus, 1758)	4	0	none	strict		larv_pred
Coleoptera	Kateretidae	*Brachypterus glaber* (Newman, 1834)	0	2	none	none	herb	
Coleoptera	Kateretidae	*Brachypterus urticae* (Fabricius, 1792)	0	3	none	none	herb	
Coleoptera	Latridiidae	*Cartodere nodifer* (Westwood, 1838)	1	0	none	none	detr	
Coleoptera	Latridiidae	*Corticaria impressa* (Olivier, 1790)	1	0	none	none	fungi	
Coleoptera	Latridiidae	*Cortinicara gibbosa* (Herbst, 1793)	2	12	none	none	fungi	
Coleoptera	Latridiidae	*Enicmus rugosus* (Herbst, 1793)	1	0	none	none	fungi	
Coleoptera	Latridiidae	*Enicmus transversus* (Olivier, 1790)	12	2	none	none	fungi	
Coleoptera	Latridiidae	*Stephostethus lardarius* (De Geer, 1775)	1	1	none	none	fungi	
Coleoptera	Leiodidae	*Choleva agilis* (Illiger, 1798)	3	0	none	none	detr	
Coleoptera	Leiodidae	*Choleva glauca* Britten, 1918	2	0	none	none	detr	
Coleoptera	Monotomidae	*Rhizophagus bipustulatus* (Fabricius, 1793)	7	0	none	none	detr	
Coleoptera	Monotomidae	*Rhizophagus dispar* (Paykull, 1800)	2	0	none	none	detr	
Coleoptera	Mycetophagidae	*Litargus connexus* (Geoffroy, 1785)	1	0	none	none	detr	
Coleoptera	Nitidulidae	*Epuraea biguttata* (Thunberg, 1784)	1	0	none	none	?	
Coleoptera	Nitidulidae	*Epurea limbata* (Fabricius, 1787)	7	0	none	none	?	
Coleoptera	Nitidulidae	*Glischrochilus hortensis* (Geoffroy, 1785)	3	0	none	none	?	
Coleoptera	Nitidulidae	*Glischrochilus quadrisignatus* (Say, 1835)	4	0	none	none	?	
Coleoptera	Nitidulidae	*Omosita colon* (Linnaeus, 1758)	5	10	strict	none		?
Coleoptera	Phalacridae	*Stilbus testaceus* (Panzer, 1796)	1	0	none	none	herb	
Coleoptera	Ptiliidae	*Acrotrichis atomaria* (De Geer, 1774)	26	0	none	none	fungi	
Coleoptera	Ptiliidae	*Acrotrichis fascicularis* (Herbst, 1793)	18	1	none	none	fungi	
Coleoptera	Ptiliidae	*Acrotrichis sitkaensis* (Motschulsky, 1845)	0	1	none	none	fungi	
Coleoptera	Ptiliidae	*Ptenidium nitidum* (Heer, 1841)	5	4	weak	weak		fungi
Coleoptera	Pyrochroidae	*Pyrochroa serraticornis* (Scopoli, 1763)	4	0	none	none	pred	
Coleoptera	Scarabaeidae	*Aphodius ater* (De Geer, 1774)	3	0	none	strict		dung
Coleoptera	Scarabaeidae	*Aphodius depressus* (Kugelann, 1792)	1	0	none	strict		dung
Coleoptera	Scarabaeidae	*Aphodius granarius* (Linnaeus, 1767)	5	0	none	strict		dung
Coleoptera	Scarabaeidae	*Aphodius luridus* (Fabricius, 1775)	180	0	none	strict		dung
Coleoptera	Scarabaeidae	*Aphodius prodromus* (Brahm, 1790)	33	0	none	strict		dung
Coleoptera	Scarabaeidae	*Aphodius sphacelatus* (Panzer, 1798)	9	0	none	strict		dung
Coleoptera	Scarabaeidae	*Onthophagus coenobita* (Herbst, 1783)	3	0	none	strict		dung
Coleoptera	Scarabaeidae	*Onthophagus vacca* (Linnaeus, 1767)	1	0	none	strict		dung
Coleoptera	Scarabaeidae	*Oxyomus sylvestris* (Scopoli, 1763)	24	0	none	weak		detr

Coleoptera	Scarptiidae	*Anaspis maculata* Geoffroy, 1785)	1	0	none	none	larv_detr	
Coleoptera	Scirtidae	*Cyphon coarctatus* Paykull, 1799	0	1	none	none	detr	
Coleoptera	Scirtidae	*Cyphon laevipennis* Tournier, 1868	5	0	none	none	detr	
Coleoptera	Silphidae	*Necrodes littoralis* (Linnaeus, 1758)	2	0	strict	none		carrion
Coleoptera	Silphidae	*Nicrophorus vespilo* (Linnaeus, 1758)	1	0	strict	none		carrion
Coleoptera	Silphidae	*Silpha tristis* Illiger, 1798	2	0	strict	none		carrion
Coleoptera	Silphidae	*Thanatophilus rugosus* (Linnaeus, 1758)	3702	0	strict	none		carrion
Coleoptera	Staphylinidae	*Acrotona aterrima* (Gravenhorst, 1802)	2	2	weak	weak		pred
Coleoptera	Staphylinidae	*Acrotona fungi* (Gravenhorst, 1806)	21	12	none	none	pred	
Coleoptera	Staphylinidae	*Acrotona orphana* (Erichson, 1837)	5	14	none	none	pred	
Coleoptera	Staphylinidae	*Acrotona pygmaea* (Gravenhorst, 1802)	1	0	none	none	pred	
Coleoptera	Staphylinidae	*Aleochara bipustulata* (Linnaeus, 1760)	1	0	weak	weak		carrionpara
Coleoptera	Staphylinidae	*Aleochara lanuginosa* Gravenhorst, 1802	9	0	weak	weak		carrionpara
Coleoptera	Staphylinidae	*Aleochara ruficornis* Gravenhorst, 1802	2	0	none	none	pred	
Coleoptera	Staphylinidae	*Aloconota gregaria* (Erichson, 1839)	29	1	none	none	pred	
Coleoptera	Staphylinidae	*Amarochara forticornis* (Lacordaire, 1835)	5	0	none	none	pred	
Coleoptera	Staphylinidae	*Amischa analis* (Gravenhorst, 1802)	12	74	none	none	pred	
Coleoptera	Staphylinidae	*Amischa bifoveolata* (Mannerhein, 1830)	0	1	none	none	pred	
Coleoptera	Staphylinidae	*Amischa decipiens* (Sharp, 1869)	0	73	none	none	pred	
Coleoptera	Staphylinidae	*Amischa forcipata* Mulsant & Rey, 1873	0	2	none	none	pred	
Coleoptera	Staphylinidae	*Amischa nigrofusca* (Stephens, 1832)	1	9	none	none	pred	
Coleoptera	Staphylinidae	*Amischa* sp.	0	3	none	none	pred	
Coleoptera	Staphylinidae	*Anotylus rugosus* (Fabricius, 1775)	129	0	none	none	detr	
Coleoptera	Staphylinidae	*Anotylus sculpturatus* (Gravenhorst, 1806)	11	0	weak	weak		detr
Coleoptera	Staphylinidae	*Anotylus tetracarinatus* (Block, 1799)	17	0	weak	weak		detr
Coleoptera	Staphylinidae	*Atheta atramentaria* (Gyllenhal, 1810)	29	0	weak	strict		pred
Coleoptera	Staphylinidae	*Atheta longicornis* (Gravenhorst, 1802)	3	0	weak	weak		pred
Coleoptera	Staphylinidae	*Atheta triangulum* (Kraatz, 1856)	4	0	weak	weak		pred
Coleoptera	Staphylinidae	*Atheta xanthopus* (Thomson, 1856)	1	0	none	none	pred	
Coleoptera	Staphylinidae	*Bisnius cephalotes* (Gravenhorst, 1802)	2	1	strict	none		pred
Coleoptera	Staphylinidae	*Bisnius fimetarius* (Gravenhorst, 1802)	1	0	weak	weak		carrion
Coleoptera	Staphylinidae	*Bisnius sordidus* (Gravenhorst, 1802)	1	12	weak	none		carrion
Coleoptera	Staphylinidae	*Callicerus obscurus* Gravenhorst, 1802	14	0	none	none	pred	
Coleoptera	Staphylinidae	*Carpelimus corticinus* (Gravenhorst, 1806)	2	0	none	none	detr	
Coleoptera	Staphylinidae	*Carpelimus elongatulus* (Erichson, 1839)	10	1	none	none	detr	
Coleoptera	Staphylinidae	*Coprophilus striatulus* (Fabricius, 1793)	3	0	none	none	detr	
Coleoptera	Staphylinidae	*Coryphium angusticolle* (Stephens, 1834)	1	0	none	none	?	
Coleoptera	Staphylinidae	*Creophilus maxilosus* (Linnaeus, 1758)	2	0	strict	none		pred
Coleoptera	Staphylinidae	*Cypha* sp.	0	1	none	none	pred	
Coleoptera	Staphylinidae	*Dinaraea aequata* (Erichson, 1837)	3	0	none	none	pred	
Coleoptera	Staphylinidae	*Dinaraea angustula* (Gyllenhal, 1810)	30	0	none	none	pred	

Coleoptera	Staphylinidae	*Dinaraea linearis* (Gravenhorst, 1802)	1	0	none	none	pred	
Coleoptera	Staphylinidae	*Drusilla canaliculata* (Fabricius, 1787)	1	0	none	none	pred	
Coleoptera	Staphylinidae	*Euaesthetus bipunctatus* (Ljungh, 1804)	18	0	none	none	pred	
Coleoptera	Staphylinidae	*Gabrius appendiculatus* Sharp, 1910	3	0	none	none	pred	
Coleoptera	Staphylinidae	*Gabrius breviventer* (Sperk, 1835)	6	0	none	none	pred	
Coleoptera	Staphylinidae	*Gabrius osseticus* (Kolenati, 1846)	34	1	none	none	pred	
Coleoptera	Staphylinidae	*Gabrius splendidulus* (Gravenhorst, 1802)	1	0	none	none	pred	
Coleoptera	Staphylinidae	*Gabrius*sp.	2	1	none	none	pred	
Coleoptera	Staphylinidae	*Geostiba circellaris* (Gravenhorst, 1806)	9	6	none	none	pred	
Coleoptera	Staphylinidae	*Gyrohypnus angustatus* Stephens, 1833	23	1	none	none	pred	
Coleoptera	Staphylinidae	*Gyrohypnus fracticornis* (Müller, 1776)	0	1	weak	weak		pred
Coleoptera	Staphylinidae	*Heterothops dissimilis* (Gravenhorst, 1802)	1	4	none	none	detr	
Coleoptera	Staphylinidae	*Ilyobates bennetti* Donisthorpe, 1914	2	0	none	none	pred	
Coleoptera	Staphylinidae	*Ischnosoma splendidum* (Gravenhorst, 1806)	1	0	none	none	pred	
Coleoptera	Staphylinidae	*Lathrobium brunnipes* (Fabricius, 1793)	16	0	none	none	pred	
Coleoptera	Staphylinidae	*Lathrobium fulvipenne* (Gravenhorst, 1806)	26	0	none	none	pred	
Coleoptera	Staphylinidae	*Lathrobium geminum* Kraatz, 1857	4	0	none	none	pred	
Coleoptera	Staphylinidae	*Lathrobium pallidipenne* Hochhuth, 1851	2	0	none	none	pred	
Coleoptera	Staphylinidae	*Lesteva longoelytrata* (Goeze, 1777)	1	0	none	none	pred	
Coleoptera	Staphylinidae	*Liogluta microptera* Thomson, 1867	5	0	none	none	pred	
Coleoptera	Staphylinidae	*Meotica pallens* (Redtenbacher, 1849)	1	0	none	none	pred	
Coleoptera	Staphylinidae	*Myllaena elongata* (Matthews, 1838)	1	0	none	none	pred	
Coleoptera	Staphylinidae	*Ocypus fuscatus* (Gravenhorst, 1802)	1	0	none	none	pred	
Coleoptera	Staphylinidae	*Ocyusa picina* (Aubé, 1750)	1	0	none	none	pred	
Coleoptera	Staphylinidae	*Oligota pusillima* (Gravenhorst, 1806)	15	38	none	none	pred	
Coleoptera	Staphylinidae	*Omalium caesum* Gravenhorst, 1806	1	0	none	none	pred	
Coleoptera	Staphylinidae	*Omalium rivulare* (Paykull, 1789)	6	0	weak	weak		pred
Coleoptera	Staphylinidae	*Othius punctulatus* (Goeze, 1777)	2	0	none	none	pred	
Coleoptera	Staphylinidae	*Oxypoda acuminata* (Stephens, 1832)	4	0	none	weak		pred
Coleoptera	Staphylinidae	*Oxypoda brachyptera* (Stephens, 1832)	146	1	none	none	pred	
Coleoptera	Staphylinidae	*Oxypoda exoleta* Erichson, 1839	134	23	none	none	pred	
Coleoptera	Staphylinidae	*Oxypoda praecox* Erichson, 1839	1	0	none	none	pred	
Coleoptera	Staphylinidae	*Oxytelus laqueatus* (Marsham, 1802)	4	0	none	strict		pred
Coleoptera	Staphylinidae	*Paederus riparius* (Linnaeus, 1858)	32	0	none	none	pred	
Coleoptera	Staphylinidae	*Parocyusa longitarsis* (Erichson, 1839)	2	0	none	none	pred	
Coleoptera	Staphylinidae	*Philhygra elongatula* (Gravenhorst, 1802)	3	0	none	none	pred	
Coleoptera	Staphylinidae	*Philhygra palustris* (Kiesenwetter, 1844)	4	0	none	none	pred	
Coleoptera	Staphylinidae	*Philonthus carbonarius* (Gravenhorst, 1802)	10	0	none	none	pred	
Coleoptera	Staphylinidae	*Philonthus cognatus* Stephens, 1832	71	0	none	none	pred	
Coleoptera	Staphylinidae	*Philonthus decorus* (Gravenhorst, 1802)	1	0	none	none	pred	
Coleoptera	Staphylinidae	*Philonthus intermedius* (Lacordaire, 1835)	1	0	weak	weak		pred

Coleoptera	Staphylinidae	*Philonthus laminatus* (Creutzer, 1799)	11	0	none	weak		pred
Coleoptera	Staphylinidae	*Philonthus politus* (Linnaeus, 1758)	4	0	strict	none		pred
Coleoptera	Staphylinidae	*Philonthus splendens* (Fabricius, 1793)	3	0	weak	weak		pred
Coleoptera	Staphylinidae	*Philonthus succicola* Thomson, 1860	1	0	strict	none		pred
Coleoptera	Staphylinidae	*Philonthus varians* (Paykull, 1889)	5	0	weak	weak		pred
Coleoptera	Staphylinidae	*Phloeocharis subtilissima* Mannerheim, 1830	9	0	none	none	?	
Coleoptera	Staphylinidae	*Platystethus alutaceus* Thomson, 1861	4	0	none	none	detr	
Coleoptera	Staphylinidae	*Platystethus arenarius* (Geoffroy, 1785)	5	1	weak	none		detr
Coleoptera	Staphylinidae	*Quedius cinctus* (Paykull, 1790)	1	0	none	none	pred	
Coleoptera	Staphylinidae	*Reichenbachia juncorum* (Leach, 1817)	3	0	none	none	pred	
Coleoptera	Staphylinidae	*Rugilus erichsonii* (Fauvel, 1867)	0	1	none	none	pred	
Coleoptera	Staphylinidae	*Rugilus rufipes* Germar, 1836	4	0	weak	none		pred
Coleoptera	Staphylinidae	*Scaphisoma agaricinum* (Linnaeus, 1758)	1	0	none	none	?	
Coleoptera	Staphylinidae	*Sepedophilus marshami* (Stephens, 1832)	13	0	none	none	?	
Coleoptera	Staphylinidae	*Sepedophilus testaceus* (Fabricius, 1793)	3	0	none	none	?	
Coleoptera	Staphylinidae	*Stenus bimaculatus* Gyllenhal, 1810	3	0	none	none	pred	
Coleoptera	Staphylinidae	*Stenus brunnipes* Stephens, 1833	9	0	none	none	pred	
Coleoptera	Staphylinidae	*Stenus canaliculatus* Gyllenhal, 1827	2	0	none	none	pred	
Coleoptera	Staphylinidae	*Stenus clavicornis* (Scopoli, 1763)	12	1	none	none	pred	
Coleoptera	Staphylinidae	*Stenus fulvicornis* Stephens, 1833	2	0	none	none	pred	
Coleoptera	Staphylinidae	*Stenus pallipes* Gravenhorst, 1802	2	0	none	none	pred	
Coleoptera	Staphylinidae	*Tachinus corticinus* Gravenhorst, 1802	15	0	none	none	pred	
Coleoptera	Staphylinidae	*Tachinus laticollis* Gravenhorst, 1802	21	0	weak	strict		pred
Coleoptera	Staphylinidae	*Tachinus rufipes* (Linnaeus, 1758)	41	0	none	weak		pred
Coleoptera	Staphylinidae	*Tachyporus atriceps* Stephens, 1832	1	0	none	none	pred	
Coleoptera	Staphylinidae	*Tachyporus dispar* (Paykull, 1789)	67	37	none	none	pred	
Coleoptera	Staphylinidae	*Tachyporus hypnorum* (Fabricius, 1775)	3	3	none	none	pred	
Coleoptera	Staphylinidae	*Tachyporus nitidulus* (Fabricius, 1781)	11	21	none	none	pred	
Coleoptera	Staphylinidae	*Tachyporus obtusus* (Linnaeus, 1767)	7	4	none	none	pred	
Coleoptera	Staphylinidae	*Tachyporus pusillus* Gravenhorst, 1806	5	16	none	none	pred	
Coleoptera	Staphylinidae	*Tachyporus* sp.	0	2	none	none	pred	
Coleoptera	Staphylinidae	*Xantholinus laevigatus* Jacobsen, 1849	1	0	none	none	pred	
Coleoptera	Staphylinidae	*Xantholinus longiventris* Heer, 1839	14	2	none	none	pred	
Coleoptera	Trogidae	*Trox scaber* (Linnaeus, 1767)	11	0	strict	none		carrion
Diptera	Calliphoridae	Maggots	4255	0	strict	none		carrion
