# Supplementary material for: Rewilding with large herbivores: Positive direct and delayed effects of carrion on plant and arthropod communities
Source: PLoS One. 2020 Jan 22;15(1):e0226946. doi: 10.1371/journal.pone.0226946 (PMC6975527; doi:10.1371/journal.pone.0226946)
Supplement: S3 Table — (PDF) [file pone.0226946.s007.pdf]

S3 Table. Summary statistic of models testing differences in species numbers between carrion and control sites. Significant effects (*P* < 0.05) are denoted bold.

Season	Functional group	Estimate	Standard error	z	p-value	power
Spring	All arthropods	0.063	0.074	0.850	0.395	0.083
Spring	Carrion-associated species	0.867	0.184	4.703	**0.000**	0.829
Spring	Carnivores	-0.021	0.080	-0.262	0.793	0.053
Spring	Herbivores	-0.066	0.153	-0.431	0.667	0.058
Spring	Detritivores	0.066	0.121	0.546	0.585	0.064
Spring	Dung-associated species	0.353	0.170	2.080	**0.038**	0.254
Summer	All arthropods	0.972	0.218	4.450	**0.000**	0.787
Summer	Carrion-associated species	Model convergence failed				
Summer	Carnivores	1.014	0.130	7.797	**<0.000**	0.998
Summer	Herbivores	0.693	0.253	2.737	**0.006**	0.399
Summer	Detritivores	2.410	0.994	2.423	**0.015**	0.327
Summer	Dung-associated species	Model convergence failed				
